# The Effectiveness of Electronic Differential Diagnoses (DDX) Generators: A Systematic Review and Meta-Analysis

**DOI:** 10.1371/journal.pone.0148991

**Published:** 2016-03-08

**Authors:** Nicholas Riches, Maria Panagioti, Rahul Alam, Sudeh Cheraghi-Sohi, Stephen Campbell, Aneez Esmail, Peter Bower

**Affiliations:** 1 NIHR Greater Manchester Primary Care Patient Safety Translational Research Centre (Greater Manchester PSTRC), Williamson Building, The University of Manchester, Manchester, United Kingdom; 2 NIHR School for Primary Care Research, Centre for Primary Care, Institute of Population Health, University of Manchester, Manchester, United Kingdom; University of Utah Health Sciences Center and ARUP Laboratories, UNITED STATES

## Abstract

**Background:**

Diagnostic errors are costly and they can contribute to adverse patient outcomes, including avoidable deaths. Differential diagnosis (DDX) generators are electronic tools that may facilitate the diagnostic process.

**Methods and Findings:**

We conducted a systematic review and meta-analysis to investigate the efficacy and utility of DDX generators. We undertook a comprehensive search of the literature including 16 databases from inception to May 2015 and specialist patient safety databases. We also searched the reference lists of included studies. Article screening, selection and data extraction were independently conducted by 2 reviewers. 36 articles met the eligibility criteria and the pooled accurate diagnosis retrieval rate of DDX tools was high with high heterogeneity (pooled rate = 0.70, 95% CI = 0.63 to 0.77; I^2^ = 97%, p<0.0001). DDX generators did not demonstrate improved diagnostic retrieval compared to clinicians but small improvements were seen in the before and after studies where clinicians had the opportunity to revisit their diagnoses following DDX generator consultation. Clinical utility data generally indicated high levels of user satisfaction and significant reductions in time taken to use for newer web-based tools. Lengthy differential lists and their low relevance were areas of concern and have the potential to increase diagnostic uncertainty. Data on the number of investigations ordered and on cost-effectiveness remain inconclusive.

**Conclusions:**

DDX generators have the potential to improve diagnostic practice among clinicians. However, the high levels of heterogeneity, the variable quality of the reported data and the minimal benefits observed for complex cases suggest caution. Further research needs to be undertaken in routine clinical settings with greater consideration of enablers and barriers which are likely to impact on DDX use before their use in routine clinical practice can be recommended.

## Introduction

Diagnostic error occurs when a clinician fails to make the correct diagnosis at an appropriate time or misses a diagnosis[1]. Rates of diagnostic error have been estimated at 10–15% in most areas of clinical medicine[2] and the estimated proportion of medico-legal claims against primary care doctors due to diagnostic error range between 63–72% [3, 4].

Addressing diagnostic error is complex and suggested approaches include training in diagnostic techniques for clinicians and the use of electronic diagnostic aids to augment the diagnostic abilities of doctors[5, 6]. Causes of diagnostic error are numerous but suggested solutions include training in diagnostic techniques for clinicians and the use of electronic diagnostic aids to augment the diagnostic abilities of doctors [6].

Differential diagnosis (DDX) generators are one form of electronic diagnostic aid and were developed in the 1960s[7]. These computer programmes suggest differential diagnoses based on clinical data input by users and the programmes vary in their computational methods such as utilising Bayesian probabilities and/or utilising text mining techniques. DDX programmes continue to evolve with their computational methods, particularly across medical specialities [8]. Some of the contemporary DDX generators available for generalist clinicians are capable of searching large electronic databases and are predominantly web-based providing easy access and flexibility in use while being continuously updated to reflect current evidence.

In one recent study, Bond and colleagues performed a head-to-head evaluation of four DDX generators which used clinical cases to rank them according to a set of criteria, with ISABEL and DxPlain scoring joint first in identifying the correct diagnosis[9]. One previous systematic review by Garg et. al reported improvements in practitioner performance following the use of disease and symptom-specific DDX generators[10]. In a more recent broader narrative review on the applications of information technology to the diagnostic process, El-Kareh and colleagues reported accuracy rates for DDX generators in the range of 70–95% and suggested the development of alternative metrics to measure diagnostic performance[11].

An awareness that DDX generators could help to address diagnostic error has been growing, including in a Kings Fund report into diagnostic error[12]. A scoping exercise performed prior to this review identified several studies which assessed the characteristics of DDX generators. Most studies reported ‘diagnostic accuracy’ as the primary outcome. This is not analogous to conventional definitions of test accuracy since DDX generators produce a differential diagnosis list of variable length. In this context, ‘diagnostic accuracy’ represents the proportion of searches in which the correct diagnosis appears in an output list of variable length. To emphasise this distinction we will subsequently use the term ‘accurate diagnosis retrieval’ in place of accuracy.

The literature demonstrates that accurate diagnosis retrieval alone does not predict the uptake and effectiveness of DDX generators in clinical settings. Other relevant characteristics which can have an impact on uptake and effectiveness include the specificity of the diagnostic list[9], time taken to use[13], availability and access [9], and cost-effectiveness[9].

There has been no previous systematic review of the effectiveness of DDX generators in general clinical practice. We therefore aimed to conduct a systematic review and meta-analysis to assess the clinical effectiveness of DDX generators. This was defined according to four key research questions:

1. Are DDX generators effective at retrieving accurate diagnoses?
2. Do DDX generators perform as well as clinicians?
3. Does consulting a DDX generator improve the accuracy of a clinician’s diagnostic list?
4. What are the enablers and barriers to the use of DDX generators in clinical practice?

The first three questions pertain to the efficacy of DDX generators. Their ability at retrieving accurate diagnoses was measured and compared with that of clinicians where applicable. The impact of DDX generators on the diagnostic performance was also assessed. The final question considers other factors which determine whether these tools have utility in clinical settings. Finally, we aimed to offer recommendations to researchers, policy makers and clinicians regarding the use of DDX generators in clinical practice and recommendations regarding the future research agenda in this area.

## Methods

### Protocol and registration

This review was conducted and reported according to Preferred Reporting Items for Systematic Reviews and Meta-analyses Statement (PRISMA) guidelines[14] (S1 PRISMA Checklist.) and registered with PROSPERO in March 2014.

Available at: http://www.crd.york.ac.uk/PROSPERO/display_record.asp?ID=CRD42014007638)

### Eligibility criteria

We included any primary research study investigating the effects of DDX generators on patient care and reporting quantitative data on pre-specified outcomes. Eligible study designs included randomised controlled trials, interrupted time series analysis, cohort studies, case control studies, cross-sectional studies and before and after studies. No language restrictions were applied.

### Exclusion criteria

DDX tools with a focus on a particular disease or speciality were excluded to minimise heterogeneity as well as their applicability for generalist clinicians as demonstrated by our scoping exercise.

### Participants

There were two groups: the individual user of the tool and the clinical case being entered into the tool. No restrictions were made on the characteristics of individual users of DDX generators, although data on training and clinical setting were recorded to allow subgroup analysis.

Cases entered by these users could be either real clinical cases or simulated cases originating from primary or secondary care, provided they were written by clinical experts and contained diagnostic uncertainty. Cases from both paediatric and adult medical specialties were included.

### Intervention

The intervention was use of a DDX generator to improve diagnostic performance. We adopted a definition of DDX generators as: “*programs which assist healthcare professionals in clinical decision making by generating a DDX based on a minimum of two items of patient data*”.

In order to be as comprehensive as possible, we included DDX generator tools which are no longer available.

### Comparator

Exploratory work identified different comparators used to determine the effectiveness of DDX generators. The following were included in this review:

1. ‘Clinical diagnosis’–used for real cases when the actual diagnosis made in practice (e.g. discharge diagnosis) is used as a proxy for gold standard diagnosis
2. ‘Simulated diagnosis’–used for cases written by a panel of experts, when the consensus opinion regarding the correct diagnosis is taken as the gold standard.
3. ‘Before’ groups—in these studies the accuracy of clinician diagnosis is compared before and after using a DDX generator.

### Outcomes

We built on previously developed criteria [15]and identified additional utility variables of relevance and extracted data on these. These are listed and defined in Table 1.

**Table 1:** Description of efficacy and utility variables.

VARIABLE	DEFINITION
**Accurate diagnosis retrieval**	*Proportion of DX tool differential lists which contain the correct diagnosis*
**Diagnostic detail -**Comprehensiveness	*Proportion of gold standard differential list which appears in the DDX differential list*
**Diagnostic detail—**Number of diagnoses	*Average number of diagnoses generated during each use of a DDX tool*
**Diagnostic detail—**Relevance	*Clinical appropriateness of DDX tool output*
**Diagnostic detail—**Diagnostic list	*Impact of using the DDX tool on the diagnostic list made by clinicians (e*.*g*. *adding / removing diagnoses)*
**Usage data—**Time to use	*Average time taken for a user to operate a DDX tool for a given case*
**Usage data—**Frequency of use	*How often DDX tools are used in a clinical setting*
**Usage data—**Satisfaction	*User satisfaction with DDX tools (can relate to patient management or educational benefits) / and/or relate to the likelihood of DDX tools being accepted and utilised by clinicians*
**Moderators of outcomes—**Case difficulty	*Impact of case complexity on DDX tool outcomes*
**Moderators of outcomes—**Clinical experience	*Impact of user’s clinical experience on DDX tool outcomes*
**Outcomes—**Investigation	*Impact of using DDX tools on the ordering of diagnostic investigations*
**Outcomes—**Cost-effectiveness	*Economic impact of using DDX tools in a clinical setting*

### Information sources and searches

The following databases were searched from inception to November 2013 and updated in June 2015: Ovid MEDLINE(R), Embase, CINAHL, Cochrane Database of Systematic Reviews, Cochrane Central Register of Controlled Trials, Cochrane Methodology Register, ACP Journal Club, Database of Abstracts of Reviews of Effects, Health Technology Assessment, NHS Economic Evaluation Database, AMED (Allied and Complementary Medicine), CAB Abstracts, Global Health, Health and Psychosocial Instruments, Health Management Information Consortium and PsycINFO. A combination of MeSH terms and text words were used describing medical diagnosis, including electronic diagnosis and the names of specific DDX generators which had been identified from exploratory work.

In addition to the websites identified by the search, hand-searches of the websites of the National Patient Safety Agency (NPSA) and the Agency for Healthcare Research and Quality (AHRQ) were undertaken. The reference lists of included articles were also screened for eligible papers and we conducted Scopus searches for all articles citing the included studies.

The complete search strategy is available in S1 File. The search was not restricted by date, language or country of publication.

### Study selection

A two-stage data selection process was followed. 1) Titles and abstracts were screened and 2) full-texts of the eligible titles and abstracts were retrieved and reviewed against the eligibility criteria. Both stages were independently completed by two reviewers and any disagreements were resolved in group meetings until consensus was reached. High inter-rater reliability was achieved: Cohen’s[16] unweighted κ coefficient = 0.88 and 0.91 for title/abstract and full-text screening, respectively.

### Data extraction

A standardised data extraction form was developed and piloted. Studies meeting the inclusion criteria were then double-extracted by the review team. The first author extracted data from all of the included studies to ensure consistency. Data were extracted and cross-checked by pairs of reviewers using the Microsoft Excel data extraction form. Disagreements were resolved by discussion in group meetings.

Participants—Extracted data on user characteristics included the total number of users in each study, clinical background, clinical grade and whether they had been trained to use the DDX generator. Case characteristics included the type of case (e.g. real vs. simulated), clinical specialty, setting (e.g. primary or secondary care), the number of unique clinical cases included in each study and the combined number of clinical cases in each study (since one case could be used by numerous users in a single study).

Intervention—We extracted data related to the type of DDX generator used, whether a complete or abbreviated list of differential diagnoses was analysed and whether it was used in real-time (e.g. prospectively or retrospectively).

We extracted data on the type of comparator used (e.g. gold standard or a ‘before’ group) as well as the type of gold standard diagnosis being used (e.g. published case report diagnosis or discharge diagnosis).

Outcomes—For each study we extracted any available data for the outcomes listed in Table 1.

### Risk of bias in individual studies

An adapted version of the Quality Assessment of Diagnostic Accuracy Studies 2 (QUADAS-2) was used to assess the methodological quality of the studies included in the review (S1 File)[17]. QUADAS-2 is specifically designed for the quality appraisal of diagnostic studies. It usually consists of seven domains; four relate to risk of bias and three relate to applicability. The scoping review highlighted several studies which had been funded by the software manufacturers which we identified as a potential source of bias. We therefore chose to incorporate an additional domain of “commercial funding” within our adapted version of QUADAS-2.

Suggested ‘signalling questions’ for each domain were tailored to this study by the authors (S1 File). Each domain was then scored as high, low or unclear. Studies were not excluded from the review on the basis of quality, but the results of the methodological quality assessment were used in the interpretation of the results.

### Data synthesis and analysis

Accurate diagnosis retrieval was the primary outcome of this review. The majority of the studies (n = 21 out of 33) reported this as a crude proportion without incorporating control groups. Rates of accurate diagnosis retrieval of the DDX generators across the studies were extracted and pooled using the single-group mode of the Comprehensive Meta-Analysis (CMA) version 2.23[18]. Subgroup analyses were performed to examine whether different types of DDX generators were associated with different levels of accurate diagnosis retrieval. We also conducted sensitivity analyses to examine whether the results altered when studies with high methodological quality ratings (based on QUADAS-2) and when DDX generators that are currently commercially available were retained in the analyses.

Two-group meta-analyses were undertaken for 7 studies which compared the accurate diagnosis retrieval rates of the DDX generators with alternative diagnostic approaches (i.e., clinical diagnoses by doctors and students) and 5 studies which examined accurate diagnosis retrieval rates before and after the use of DDX generators (before and after studies; n = 5)[19–23]. Accurate diagnosis retrieval data from these two groups of studies were extracted and converted into a common effect size (Standardised Mean Difference; SMD) and pooled in CMA. A positive SMD indicated that DDX generators were associated with higher levels of accurate diagnosis retrieval whilst a negative SMD indicated that DDX generators were associated with lower levels of accurate diagnosis retrieval [24]. In keeping with established cut-off points of effect, effect sizes of 0.7 and higher were categorised as large; effect sizes of 0.30 to 0.60 as moderate, and effect sizes 0.2 and lower as small[25]. A random effects model was used throughout to control for between-study heterogeneity. STATA software (version 13) was used to create the forest plots.

The Cochran’s Q statistic[26] and the Higgin’s I^2^ [26]statistic were used to assess between-study heterogeneity. The Q statistic provides an estimate of whether differences between meta-analysed studies are greater than would be expected by chance. Statistically significant results indicate the presence of heterogeneity. The I^2^ statistic provides a quantitative measure of the degree of between study differences caused by factors other than sampling error. Higher I^2^ values represent greater heterogeneity[26]. Publication bias was examined using a test of funnel plot asymmetry (Egger’s test)[27] and Rosenthal's fail safe N (FSN) [28]. Egger’s test reveals whether or not the funnel plot is symmetric and indicates the existence/absence of a significant publication bias, and the FSN provides an estimate of the number of studies with statistically non-significant results are needed for a meta-analysed finding to become statistically non-significant.

A considerably lower number of studies reported data on the utility of DDX generators compared to studies reporting data on accurate diagnosis retrieval. A wide range of outcomes were described as “utility” outcomes, however due to the low number of studies and the inconsistent reporting of data, utility outcomes were precluded from a meta-analyses. Hence, a narrative synthesis was undertaken for utility outcomes.

## Results

**Fig 1:**
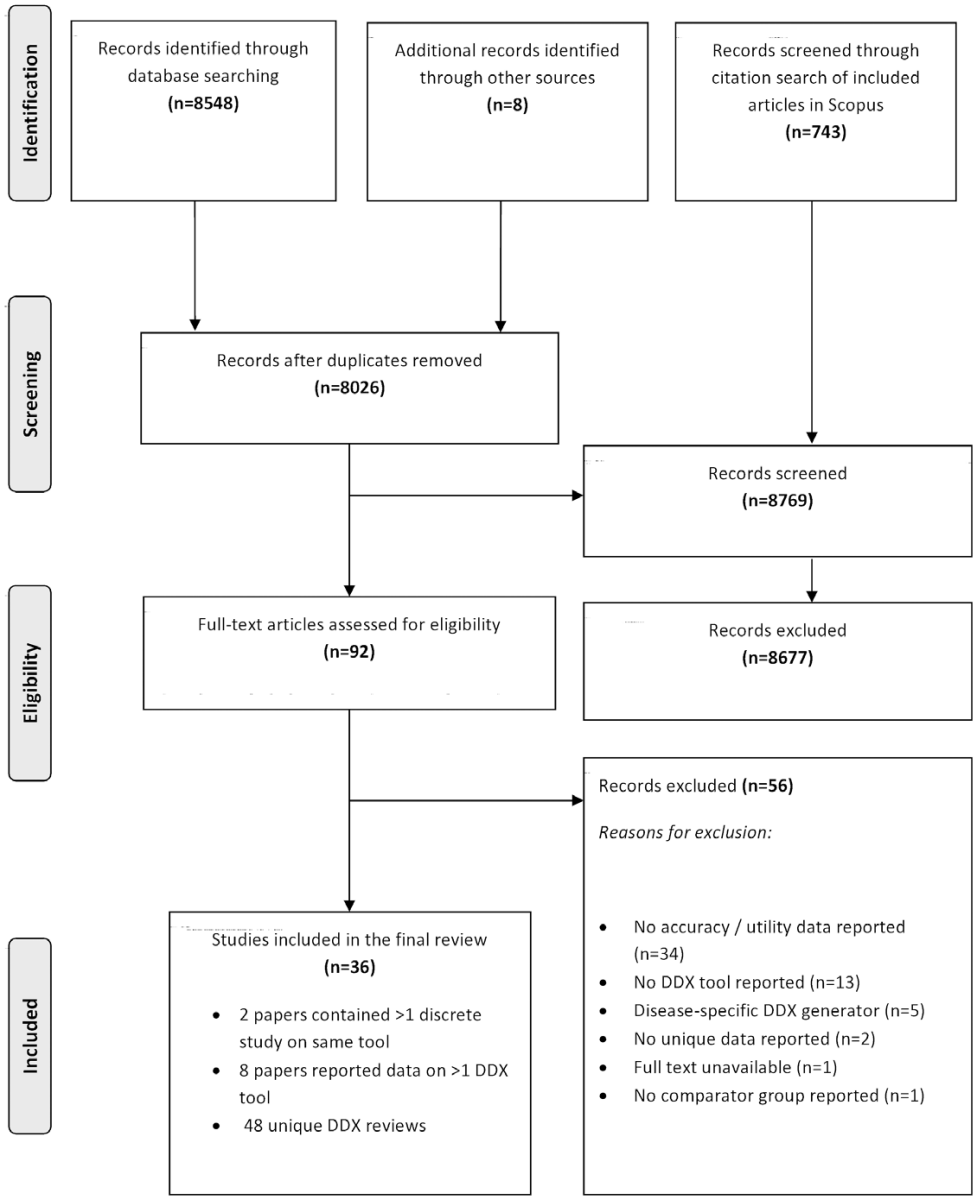
PRISMA flowchart.

The PRISMA flowchart demonstrates the screening and selection process for the review and is outlined in Fig 1. The search generated 9299 references and following initial screening, 92 articles were short-listed for full text screening. Of these, 36 articles met the eligibility criteria[9, 13, 15, 19–23, 29–56]. Two articles[46, 55] included 2 discrete studies on the same DDX generator and these were considered as separate studies for the purposes of this review leading to a total of 38 eligible studies. Additionally, 6 articles reported data on more than one DDX generator and these were also considered as separate studies[9, 13, 15, 19, 39, 51]. This resulted in a total of 48 independent DDX generator comparisons reporting either diagnostic accuracy; clinical utility data, or a combination.

### Study characteristics

Complete study characteristics are detailed in Table 2. In total, 36 articles provided data on 11 different DDX tools, of which 6 are known to be no longer commercially available.

**Table 2:** Study characteristics.

STUDY ID	INDEX TEST	COUNTRY	DESIGN	SETTING	SPECIALITY	CASE TYPE	CASES ANALYSED		INVESTIGATOR BACKGROUND	DATA ENTRY
							Unique cases	Total		
Apkon 2005[29]	PKC	USA	RCT	Primary care	General practice	Real	1902	1902	Other	Unclear
Arene 1998[30]	QMR	USA	DAS	Hospital	General medicine	Real	40	80	Clinician	Retrospective
Bacchus 1994[31]	QMR	Canada	RCT	Hospital	General medicine	Simulated	9	96	Clinician	Prospective
Bankowitz 1989[32]	QMR	USA	DAS	Hospital	General medicine	Real	20	20	Clinician	Retrospective
Bavdekar 2005[49]	ISABEL	India	DAS	Hospital	Paediatrics	Real	200	200	Unclear	Retrospective
Berner 1994[15]	DxPLAIN	USA	DAS	Hospital	General medicine	Real	63	63	Unclear	Retrospective
Berner 1994[15]	ILIAD	USA	DAS	Hospital	General medicine	Real	63	63	Unclear	Retrospective
Berner 1994[15]	MEDITEL	USA	DAS	Hospital	General medicine	Real	63	63	Unclear	Retrospective
Berner 1994[15]	QMR	USA	DAS	Hospital	General medicine	Real	63	63	Unclear	Retrospective
Berner 1999[33]	QMR	USA	DAS	Unclear	Unclear	Simulated	24	863	Clinician	Retrospective
Bond 2011[9]	ISABEL	USA	DAS	Unclear	General medicine	Real	20	20	Academic	Retrospective
Bond 2011[9]	DxPLAIN	USA	DAS	Unclear	General medicine	Real	20	20	Academic	Retrospective
Bond 2011[9]	DIAGNOSIS PRO	USA	DAS	Unclear	General medicine	Real	20	20	Academic	Retrospective
Bond 2011[9]	PEPID	USA	DAS	Unclear	General medicine	Real	20	20	Academic	Retrospective
Carlson 2011[34]	ISABEL	USA	DAS	Primary care	General practice	Simulated	4	4	Medical student	Retrospective
Elkin 2010[35]	DxPLAIN	USA	Observational study	Hospital	General medicine	Mixed	323	323	Clinician	Prospective
Elstein 1996[36]	ILIAD	USA	DAS	Hospital	General medicine	Real	36	144	Clinician	Retrospective
Feldman 1991[50]	DxPLAIN	USA	DAS	Unclear	General medicine	Mixed	46	46	N/A	Retrospective
Friedman 1999[19]	ILIAD	USA	DAS	Hospital	General medicine	Real	36	1935	Clinician	Retrospective
Friedman 1999[19]	QMR	USA	DAS	Hospital	General medicine	Real	36	1935	Clinician	Retrospective
Gozum 1994 [51]	QMR	USA	DAS	Academic	General medicine	Real	5	110	Medical student	Retrospective
Gozum 1994 [51]	ILIAD	USA	DAS	Academic	General medicine	Real	5	110	Medical student	Retrospective
Graber 2003[13]	QMR	USA	DAS	Hospital	Emergency medicine	Real	25	25	Unclear	Retrospective
Graber 2003[13]	ILIAD	USA	DAS	Hospital	Emergency medicine	Real	25	25	Unclear	Retrospective
Graber 2008[37]	ISABEL	USA	DAS	Unclear	General medicine	Real	50	50	Unclear	Retrospective
Graber 2009[38]	ISABEL	USA	DAS	Unclear	General medicine	Unclear	3	33	Medical student	Retrospective
Hammersley 1988[39]	MEDITEL	USA	DAS	Hospital	General medicine	Real	103	103	Unclear	Retrospective
Hammersley 1988[39]	DxPLAIN	USA	DAS	Hospital	General medicine	Real	103	103	Unclear	Retrospective
Heckerling 1991[20]	ILIAD	USA	DAS	Hospital	General medicine	Real	50	100	Clinician	Retrospective
Lange 1997[40]	ILIAD	USA	DAS	Unclear	General medicine	Simulated	8	72	Clinician	Retrospective
Lau 1995[52]	ILIAD	USA	DAS	Hospital	General medicine	Real	326	326	Clinician	Retrospective
Lemaire 1999[41]	QMR	Canada	DAS	Hospital	General medicine	Real	154	308	Clinician	Retrospective
Li 1995[42]	ILIAD	USA	Observational study	Hospital	General medicine	Real	20	80	Unclear	Retrospective
Lincoln 1991[53]	ILIAD	USA	DAS	Hospital	General medicine	Mixed	10	800	Medical student	Retrospective
Miller 1982[54]	INTERNIST-I	USA	DAS	Hospital	General medicine	Real	19	43	Unclear	Retrospective
Miller 1986[43]	QMR	USA	DAS	Hospital	General medicine	Real	36	36	Academic	Retrospective
Murphy 1996[21]	ILIAD	USA	DAS	Hospital	General medicine	Real	36	297	Clinician	Retrospective
Nelson 1985[44]	RECONSIDER	USA	DAS	Hospital	General medicine	Real	797	797	Academic	Retrospective
Ramnarayan& Roberts 2006[22]	ISABEL	UK	DAS	Hospital	Paediatrics	Real	24	751	Clinician	Retrospective
Ramnarayan&Winrow 2006[23]	ISABEL	UK	DAS	Hospital	Paediatrics	Real	104	104	Clinician	Prospective
Ramnarayan& Tomlinson 2003 (Pt. 1)[55]	ISABEL	UK	DAS	Hospital	Paediatrics	Simulated	99	99	Academic	Retrospective
Ramnarayan& Tomlinson 2003 (Pt. 2)[55]	ISABEL	UK	DAS	Hospital	Paediatrics	Real	87	87	Academic	Retrospective
Ramnarayan 2007[45]	ISABEL	UK	DAS	Hospital	Emergency medicine	Real	217	217	Academic	Retrospective
Rodriguez-Gonzalez 2012[56]	ML-DDSS	Spain	DAS	Unclear	General medicine	Unclear	20	60	Unclear	Retrospective
Waxman 1990 (retrospective)[46]	MEDITEL	USA	DAS	Hospital	General medicine	Real	30	30	Unclear	Retrospective
Waxman 1990 (prospective)[46]	MEDITEL	USA	DAS	Hospital	General medicine	Real	51	51	Unclear	Prospective
Wexler 1975[47]	MEDITEL	UK	DAS	Hospital	Paediatrics	Real	67	50	Unclear	Retrospective
Wolf 1997[48]	ILIAD	USA	Observational study	Hospital	General medicine	Real	136	136	Clinician	Retrospective

The majority of included studies were diagnostic accuracy studies (n = 33 discrete DDX tool investigations) comparing diagnostic accuracy using a DDX generator with a pre-determined gold standard diagnosis[9, 13, 15, 19–22, 30–34, 36–41, 43–47, 49–56]. Five articles reported no accuracy data[23, 29, 35, 42, 48] and 29 studies from 28 articles reported at least one component of utility data[13, 15, 19–23, 29–48, 55] (one article comprised of 2 separate studies containing utility data [46]).

5 articles contained control groups, 3 of which were observational studies[35, 42, 48] and 2 were randomised controlled trials[29, 31]. Summary characteristics of all the included studies are presented in Table 2.

### Risk of bias

Quality scores were variable with more recent studies demonstrating higher quality[9, 23, 29, 35, 37, 49]. The risk of bias for individual studies is listed in S2 File.

**Fig 2:**
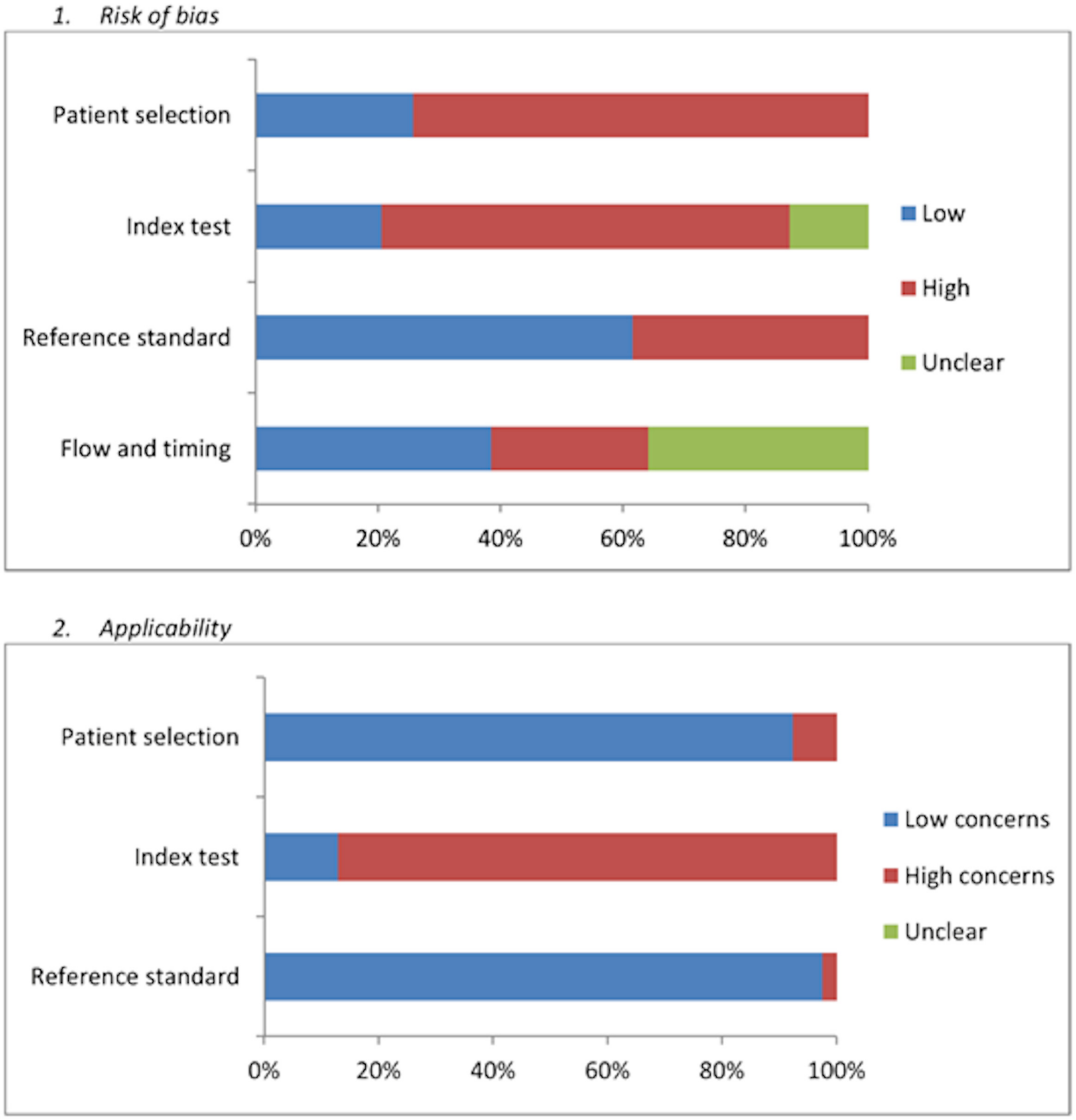
Risk of Bias summary table.

When the risk of bias was summarised across studies (Fig 2), a particularly high risk of bias was demonstrated in relation to case selection. This was typically due to a lack of randomization or inappropriate exclusions. Applicability was generally good for case selection and the reference standard. However, this was not the case for the index test given that most studies were not performed in real-time clinical settings. Only three studies received low risk of bias scores across seven or more criteria[9, 35, 57].11 studies received low risk of bias scores across 5 criteria[20, 23, 29, 31, 36, 38, 39, 41, 47–49] and the remaining studies were deemed to have a high risk of bias [13, 15, 19, 21–23, 30, 33, 34, 40, 42–46, 50–56, 58].

### Synthesis of results

Data across the vast majority of the studies reporting accurate diagnosis retrieval data (28 of 33) were included in single-group meta-analysis to examine the first research question. The majority of these studies (21 of 28) did not include a comparator; rather the correct diagnoses were confirmed a priori by expert clinician diagnoses.

Only 7 of the 33 studies compared the accurate diagnosis retrieval of DDX generators in assigning the correct diagnoses against other diagnostic methods (e.g. clinician diagnosis) in addition to the pre-assigned expert clinical diagnoses [30–32, 38, 43, 47, 56]. These 7 studies were initially included in single-group meta-analysis (n = 28) and then were further analysed in 2-group meta-analysis to examine the second research question. The remaining 5 of the 33 studies reported accurate diagnosis retrieval data before and after the use of DDX generators. A separate two-group meta-analysis was undertaken for these 5 studies to examine the third research question.

In relation to the fourth research question, data on 11 different utility outcomes representing enablers or barriers to the use of DDX generators in clinical practice were identified but these were reported inconsistently across the studies preventing a meta-analysis, therefore a narrative synthesis of these outcomes was undertaken. The DDX tool specific data on utility outcomes are presented in S2 File.

### Are DDX generators effective at retrieving accurate diagnoses?

**Fig 3:**
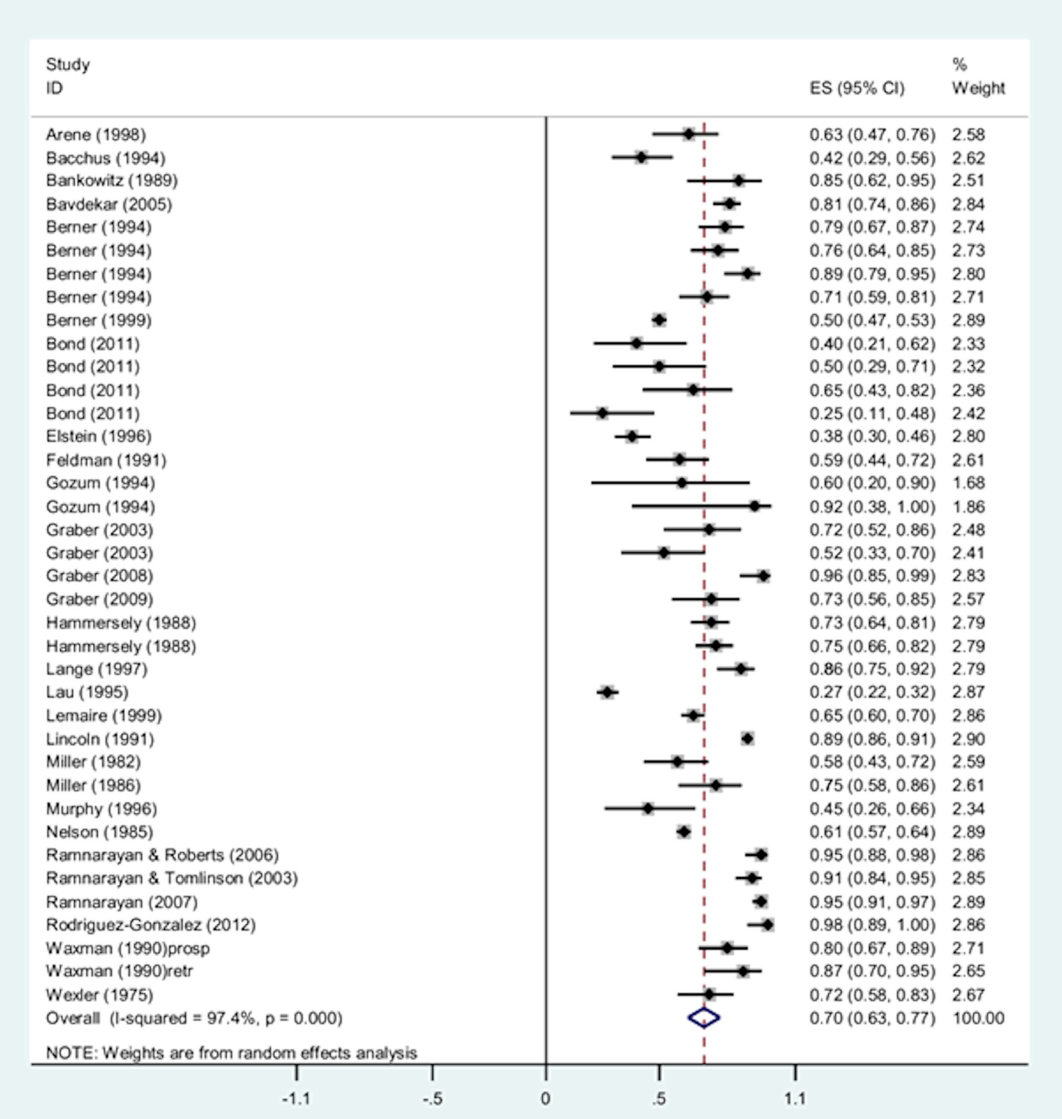
Single-group meta-analysis: Forest plot of the total accurate diagnosis retrieval rates of DDX generators. Heterogeneity chi-squared = 1414.31 (d.f. = 37), p < 0.0001. Note: Random effects model used. 95% CI = 95% confidence intervals; ES = rates.

Five of the 28 studies provided accurate diagnosis retrieval data on multiple DDX generators [9, 13, 19, 39, 51] resulting in a total of 38 independent samples included in the meta-analysis (see forest plot in Fig 3).

The pooled accurate diagnosis retrieval rate of DDX tools was high but the heterogeneity was also high (pooled rate = 0.70, 95% CI = 0.63 to 0.77; I^2^ = 97%, p<0.0001) (see Fig 3). The individual rates ranged widely from 0.25 in a study examining the accurate diagnosis retrieval rate of the PEPID generator[9] to 0.98 in a study examining the accurate diagnosis retrieval rate of the ML-DDS generator based on only 5 cases[56]. As shown in Fig 3, a total of 11 studies reported accurate diagnosis retrieval rates lower than 60 percentage points and 13 studies reported accurate diagnosis retrieval rates higher than 0.80.

#### Subgroup analysis

**Fig 4:**
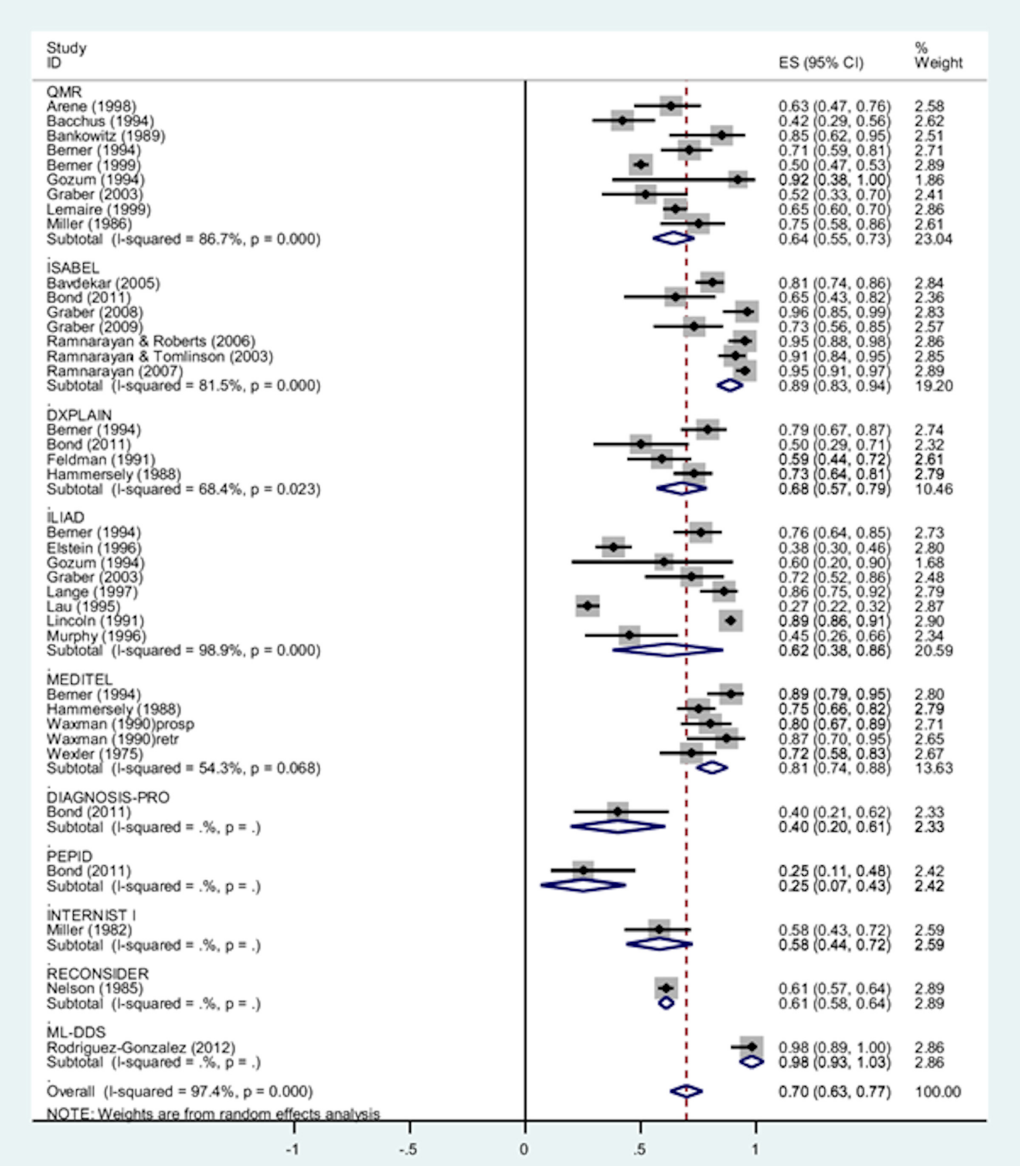
Single-group meta-analysis: Forest plot of the accurate diagnosis retrieval rates by subgroups of DDX generators. Chi-squared values: DXPLAIN = 9.64 (d.f. = 3), p = 0.023, ILIAD = 634.49 (d.f. = 7), p < 0.0001, ISABEL = 33.61 (d.f. = 6), p < 0.0001, MEDITEL = 8.73 (d.f. = 4), p = 0.068, QMR = 60.24 (d.f. = 8), p < 0.0001. Note: Random effects model used. 95% CI = 95% confidence intervals; ES = rates.

A subgroup analysis was conducted to examine whether different types of DDX generators were associated with different levels of accurate diagnosis retrieval (see forest plot in Fig 4). ISABEL[9, 22, 23, 34, 37, 38, 45, 49, 55] was associated with the highest rates of accurate diagnosis retrieval compared to all other types of DDX tools, but heterogeneity was high (pooled rate = 0.89, 95% CI = 0.83 to 0.94; I^2^ = 82%, p<0.0001). MEDITEL[39, 46, 47] was also associated with high rates of accurate diagnosis retrieval with moderate heterogeneity (pooled rate = 0.81, 95% CI = 0.74 to 0.88; I^2^ = 54%, p = 0.07). Moderate rates of accurate diagnosis retrieval were observed for DXPLAIN[9, 39, 50](pooled rate = 0.68, 95% CI = 0.57 to 0.79; I^2^ = 68%, p = 0.02) ILIAD[13, 19–21, 36, 40, 51–53] (pooled rate = 0.62, 95% CI = 0.38 to 0.86; I^2^ = 99%, p<0.0001) and QMR[13, 19, 30–33, 41, 43, 51] (pooled rate = 0.64, 95% CI = 0.55 to 0.73; I^2^ = 87%, p<0.0001) but again heterogeneity was high. In one article, accurate diagnosis retrieval rates for DIAGNOSIS PRO and PEPID were reported and demonstrated low rates of accurate diagnosis retrieval [9]. Three other types of diagnostic tools (INTERNIST-1, ML DDSS, RECONSIDER) were reported in 3 articles[44, 54, 56] but again these rates were based on the results of a single study and these tools are not commercially available.

#### Sensitivity analyses

**Fig 5:**
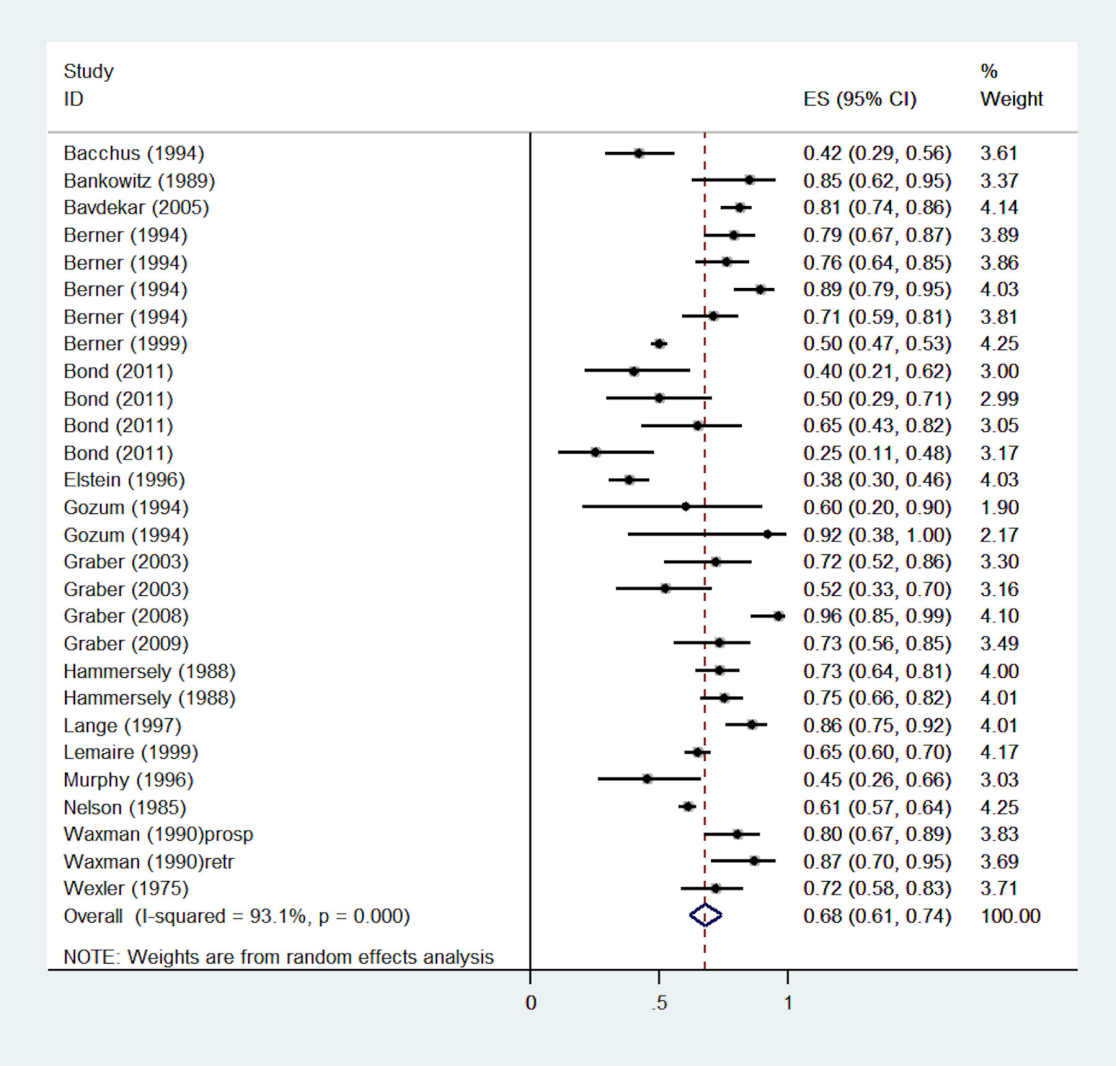
Sensitivity analysis: Forest plot of the rates of accurate diagnosis retrieval of DDX generators across studies with stronger methodological quality ratings. Heterogeneity chi-squared = 267.70 (d.f. = 13), p = 0.000. Note: Random effects model used. 95% CI = 95% confidence intervals; ES = rates.

From the pooled single-group meta-analyses we removed studies which were assigned high risk of bias ratings across 4 or more criteria. Eighteen studies including 28 independent samples were retained in the meta-analysis [9, 13, 15, 21, 32, 33, 36–41, 44, 46, 47, 49, 51]. The pooled effect size was slightly lower compared to the pooled effect size obtained in the overall analysis (pooled rate = 0.68, 95% CI = 0.61 to 0.74, I^2^ = 93.1%, p<0.0001) (see forest plot in Fig 5).

**Fig 6:**
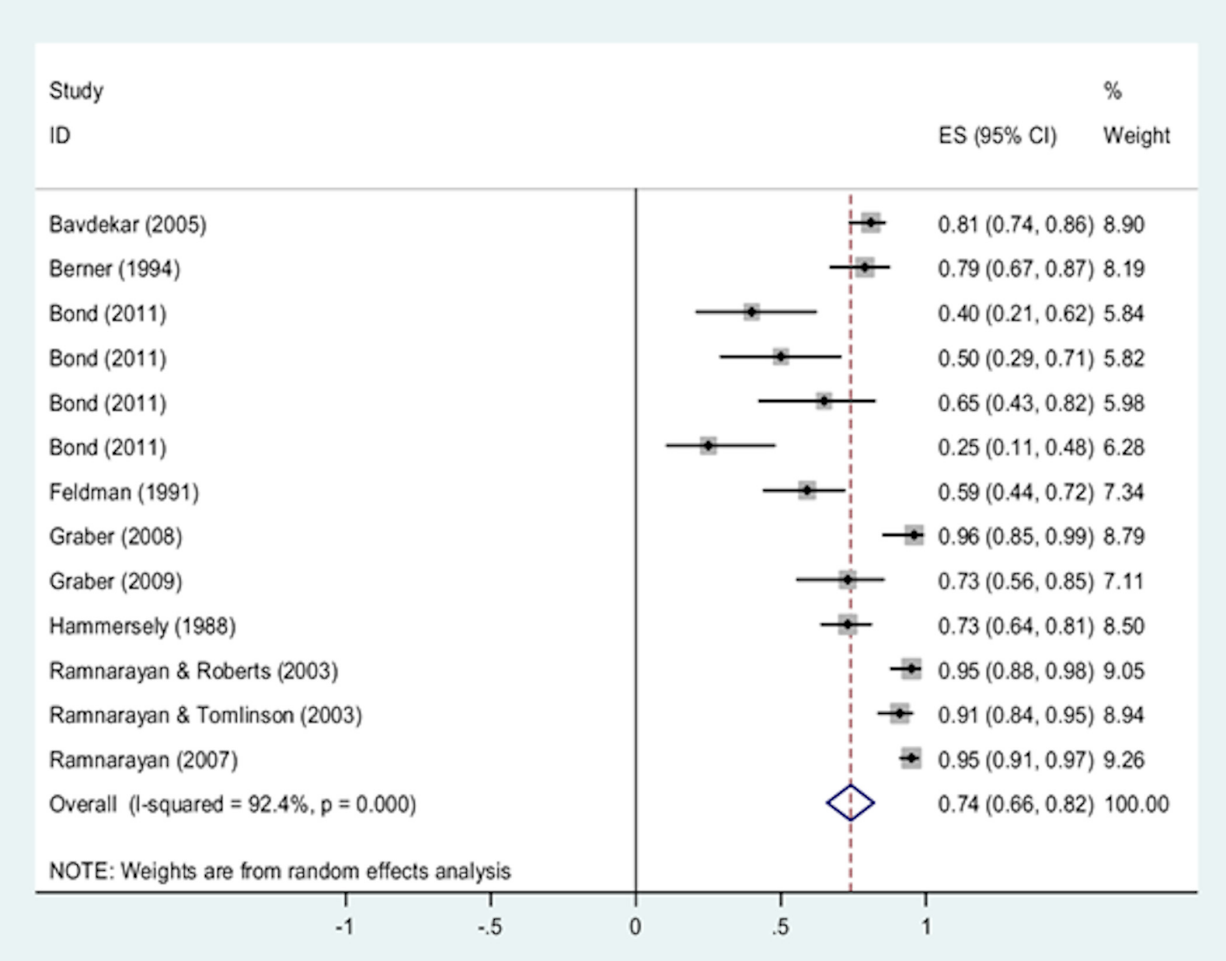
Sensitivity analysis: Forest plot of the rates of accurate diagnosis retrieval of DDX generators across studies testing commercially available DDX generators. Heterogeneity chi-squared = 158.80 (d.f. = 12), p < 0.0001. Note: Random effects model used. 95% CI = 95% confidence intervals; ES = rates.

An additional sensitivity analysis was performed in which only studies reporting commercially available DDX generators were retained in the analysis (10 studies reporting 13 independent samples) [9, 15, 22, 37–39, 45, 49, 50, 55]. A slightly increased pooled effect size was found for the commercially available DDX generators (pooled rate = 0.74, 95% CI = 0.66 to 0.82, I^2^ = 92%, p< 0.0001) compared to the pooled effect size of the main analysis (see Fig 6). This result however was largely affected by the poor accurate diagnosis retrieval rates of PEPID and Diagnosis PRO which were only reported by a single study. A substantially higher pooled rate was obtained when only the 2 commonest commercially available DDX generators were retained in the analysis (pooled rate = 0.81, 95% CI = 0.74 to 0.88, I^2^ = 89%, p< 0.0001 (data not shown).

#### Publication Bias

**Fig 7:**
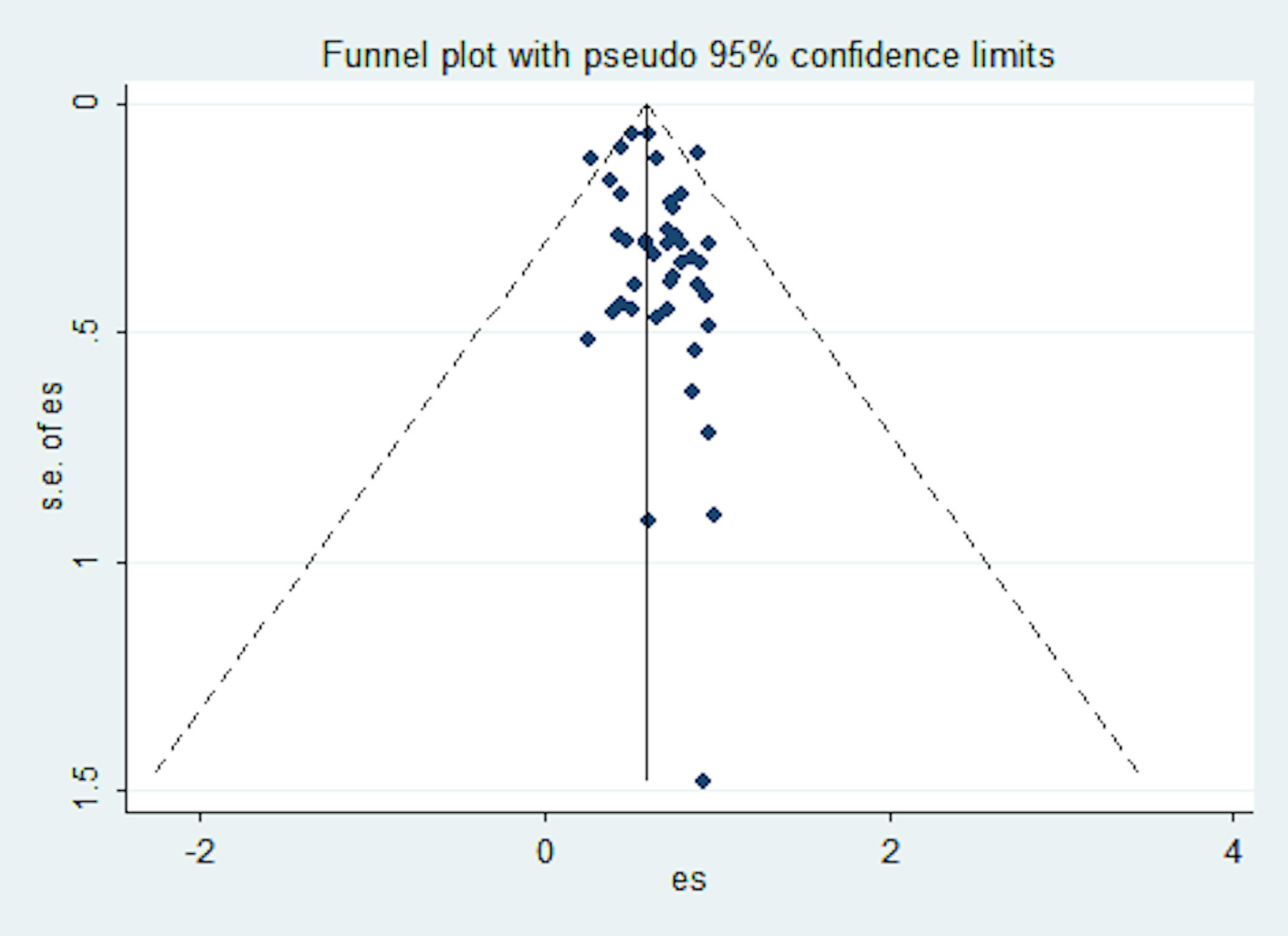
Funnel plot for studies examining the accurate diagnosis retrieval rates of DDX generators. Note: ES = rates, SE of SE = standard error of rates.

No funnel plot asymmetry (see Fig 7) was identified and Egger test was non-significant suggesting that no publication bias is present (regression intercept = 0.42, SE = 0.21, p = 0.053). Moreover, the FSN test indicated that as many as 1056 studies would be needed to nullify the significant effects obtained in the single-group meta-analysis.

### Do DDX generators perform as well as clinicians?

**Fig 8:**
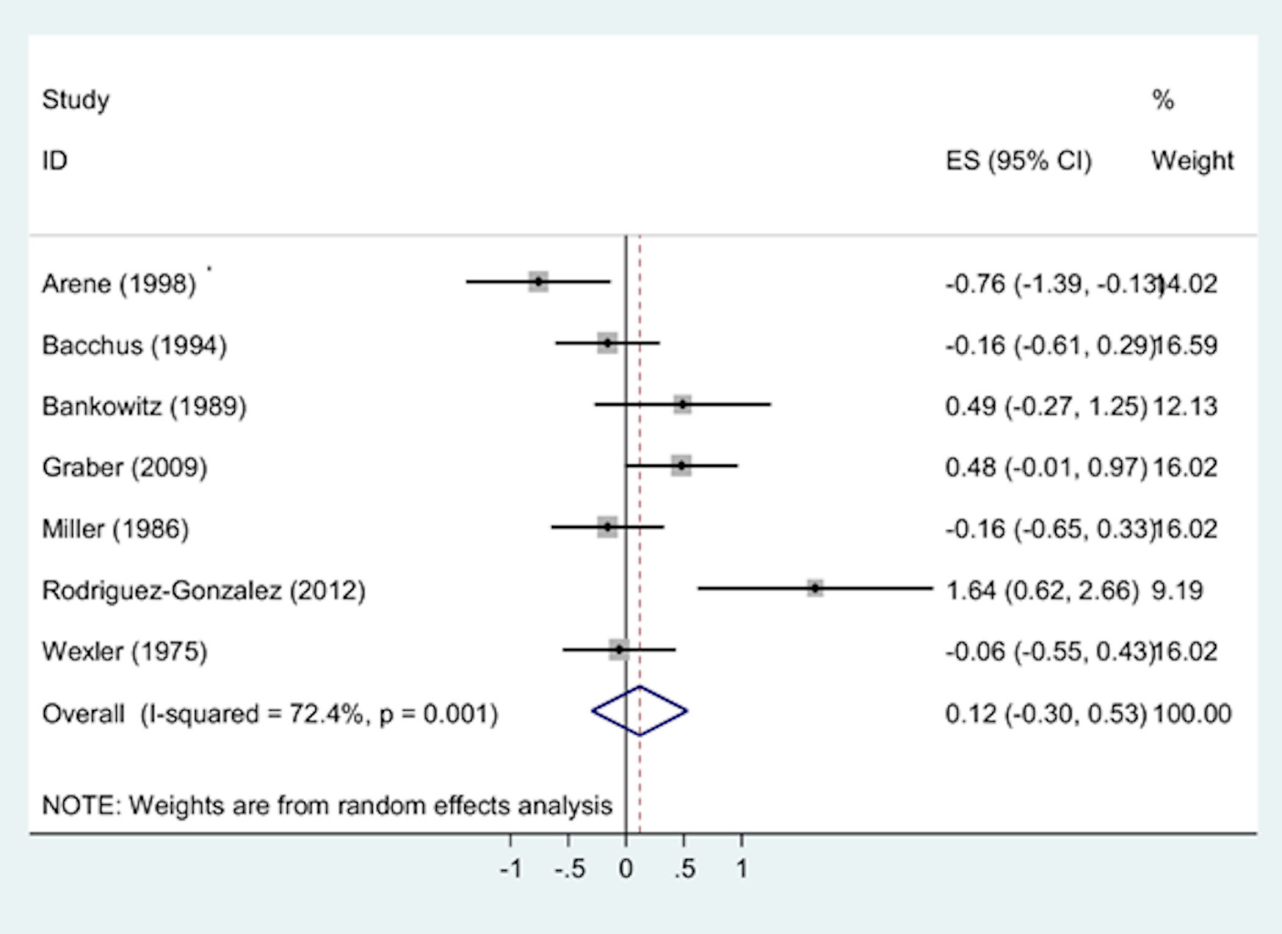
Two-groups meta-analysis: Forest plot of the accurate diagnosis retrieval rates of DDX generators compared to other types of clinical diagnoses. Heterogeneity chi-squared = 21.70 (d.f. = 6), p = 0.001. Note: Random effects model used. 95% CI = 95% confidence intervals; ES = Standardised mean difference.

The pooled standardised mean difference (SMD) of the 7 studies [30–32, 38, 43, 47, 56] which compared the efficiency of DDX tools with a comparator (clinical diagnoses by doctors (n = 6) and students (n = 1)), indicated that the use of DDX tools was associated with small, non-significant increases in accurate diagnosis retrieval compared to other ways of assigning diagnoses but the heterogeneity was high (SMD = 0.12, 95% CI = -0.30 to 0.53, I^2^ = 72%, p < 0.0001; Fig 8). ISABEL was associated with the highest accurate diagnosis retrieval rates compared to all other DDX generators[38].

### Does clinician use of a DDX generator after initial diagnosis lead to more accurate diagnoses?

**Fig 9:**
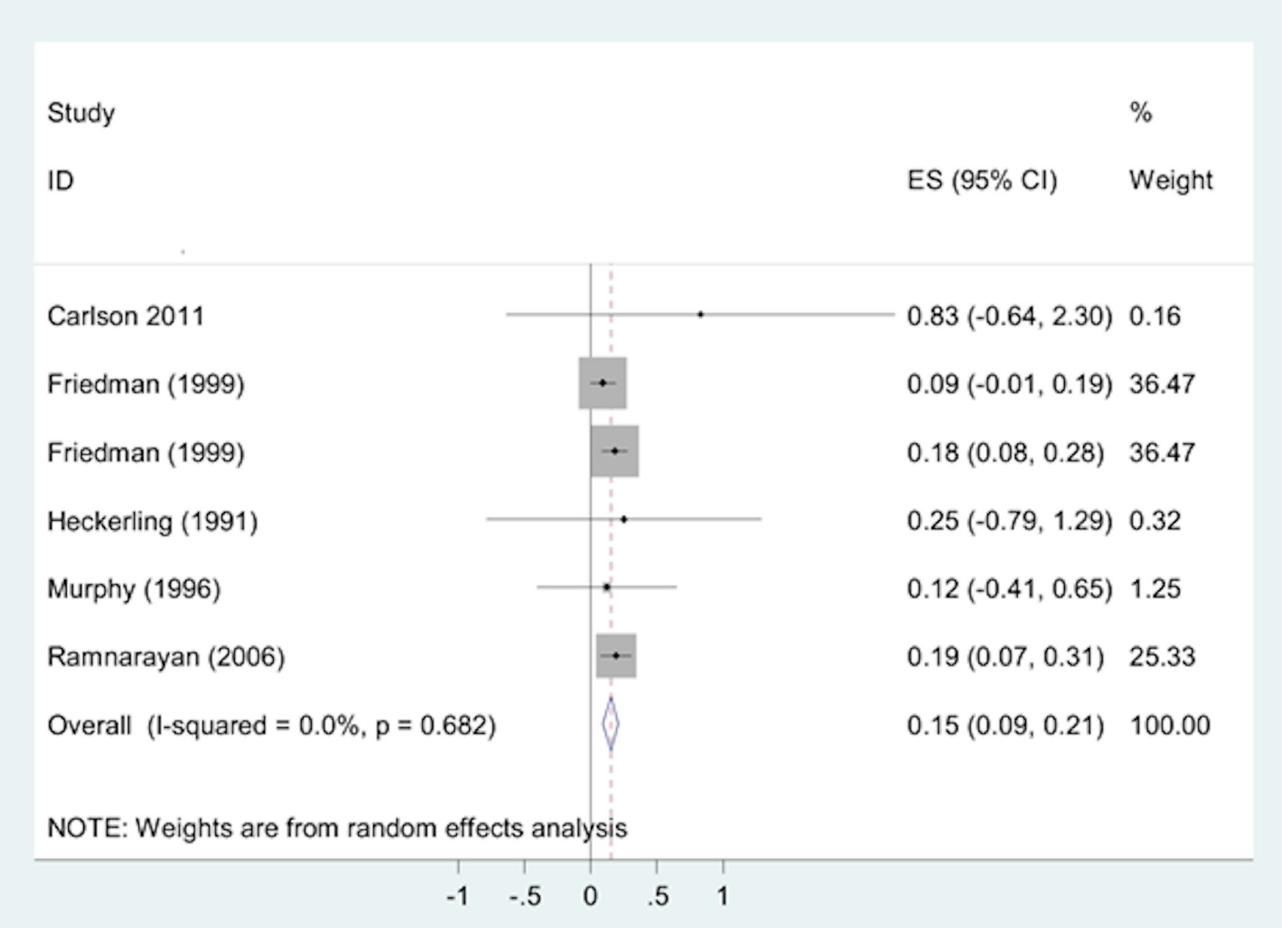
Two-groups meta-analysis: Forest plot of the accurate diagnosis retrieval rates of DDX generators in before-after studies. Heterogeneity chi-squared = 2.29 (d.f. = 4) p = 0.682. Note: Random effects model used. 95% CI = 95% confidence intervals; ES = rates.

Five studies[19–22, 34] including six independent samples examined differences in accurate diagnosis retrieval rates before and after the use of DDX tools. The pooled effects of the DDX tools indicated small, significant improvements in accurate diagnosis retrieval but the clinical significance of these minor benefits is uncertain (SMD = 0.15, 95% CI = 0.09 to 0.21; Fig 9).

### What are the enablers and barriers to the use of DDX generators in clinical practice?

The complete utility data is contained in S3 File, grouped by DDX generator tool. This section summarizes this evidence according to the previously identified utility variables.

#### Diagnostic detail

The *relevance* of a DDX list was primarily reflected by the position of the correct diagnosis in the DDX list although conventions for truncating and displaying differential lists varied across systems (see S2 File). In two studies, the correct diagnosis appeared 1^st^ in the differential list in 28%[44] and 23%[41] of cases and in 4 other studies the correct diagnosis appeared in the top 10 list for 78%[45], 68%[44], 51%[13], and 44%[13] of cases. The mean ranking of the correct diagnosis was reported in 4 studies at 2.3[20], 9^th^[39], 10^th^[46] and 10.7[39]. Additionally, one study comparing four tools framed relevance on the proportion of DDX tool-generated diagnoses felt to be appropriate by experts and reported values of 46%[15], 26%[15], 23%[15] and 21%[15]. Five others reported inconclusive and heterogeneous forms of individual study relevance scores[15, 19, 22, 33, 45].

Six studies[15, 20, 33, 41, 43, 55] reported on the *comprehensiveness* of the differential diagnosis output list across five tools and the scores varied according to the DDX generator: DxPlain = 38% (33–44%)[15]; ILIAD = 27% (22%-32%)[15] and 56%[20]; ISABEL = 87%[55]; MEDITEL = 39% (32–46%)[15]; and QMR = 30% (25–35%)[15], 22%[33] and 89%[41].

Nine studies[15, 19, 21–23, 32, 36, 42, 43] reported the impact of DDX generators on the content of user’s *diagnostic lists*. In 6 of these studies, the use of DDX tools were associated with an increased length of diagnostic list,[15, 19, 21–23, 32] one indicated a decline in the quality of the diagnostic list[42] and one indicated no impact[43]. In two studies it was reported that the correct diagnosis was prompted by the DDX generator but then ignored by clinicians[23, 36] while another study found cases where the correct diagnosis was removed following DDX generator-consultation[19] (6.3% of cases when using ILIAD and 5.8% when using QMR). Ramnarayan also found that using ISABEL led to a significant reduction in the number of incorrect diagnoses[23].

Finally, there were seven studies where data was provided on the *number of diagnoses* generated by DDX generators[30, 32, 38, 39, 41, 47, 55]. The mean number of diagnoses generated by historical tools such as MEDITEL (40.6[47] and 46.5[39]) and QMR (48.5[30]) were greater than the most recent tool, ISABEL (13[55] and 30[38])

#### Usage data

Of the 36 studies, only 3 utilised the DDX tools in real time and one of the 36 did not report when the tool was utilised. Two studies utilised the tools in primary care, 26 in hospitals, one in an academic setting and 7 studies were unclear.

There were six comparisons from five studies which reported data on the time taken to use DDX generators[22, 23, 32, 37, 39]. Historical tools ranged from 22 minutes for MEDITEL[39], 30 minutes for DxPLAIN[39] and up to 240 minutes per case episode for QMR[32]. By contrast, ISABEL was found to take between 98 seconds and six minutes on average per case in two studies[22, 23] and less than a minute in another[37].

Two studies reported data on the *frequency of use* of DDX generators and they both related to the use of the ISABEL tool. In a study by Graber, 56% of students randomized to use ISABEL to solve a clinical problem actually made use of it[38]. In a study where clinicians were given open access to ISABEL, only 7.9% reported using it more than weekly in one instance and in another study in the same paper, 54% of clinicians completed all 12 allocated cases using ISABEL[22].

Finally, seven studies reported on user *satisfaction rates* with DDX generators and they generally reported satisfaction rates to be high,[23, 30–32, 34, 43, 48] although subject to reporting bias. Some users suggested that it may be most beneficial as an educational aid for teaching diagnostic skills in a simulated environment[23, 34].

#### Moderators of outcomes

Six studies considered the *clinical experience* of clinicians as a moderator of outcomes[21–23, 34, 36, 40]. When the impact of DDX generators on accurate diagnosis retrieval was compared between different clinician grades; generally the inexperienced users such as medical students[21, 22, 34, 36] and Senior House Officers[22, 23] benefited the most. The way in which users operate the tool also appeared to vary with experience with inexperienced users inputting more data into the tool[36]. Medical students were also significantly more likely to add diagnoses to a pre-existing differential list compared with more senior doctors[21, 22].

Four studies reported on the impact of *case difficulty*[22, 30, 33, 44] and found that the accurate diagnosis retrieval rates of DDX generators are lower for complex cases than easier ones[22, 30, 33] and are also lower in cases where there are multiple diagnoses[44].

#### Outcomes

In addition to the impact of DDX generators on the diagnostic process itself, two other important outcomes which were reported related to the impact of using DDX generators on *investigation requests*, reported by four studies[22, 23, 31, 47], and on overall *cost-effectiveness*, reported by two studies[29, 35]. Ramnarayan et al. found that at least one significant investigation was added to the management plan following ISABEL consultation in 9.3% of cases[22]. In a separate investigation published in the same paper he found that the average number of tests ordered tended to increase following use of ISABEL[22]. Another study found that the quality of investigations arranged following DDX consultation did not increase[31] and one reported a decrease in the number of unnecessary investigations being ordered [47].

Apkon et al. found a significant increase in laboratory testing and total resource consumption for the group randomized to receive PKC[29]. By contrast Elkin found that total hospital admission costs were significantly reduced in a cohort of patients whose clinicians were using DxPLAIN, versus normal care, although there was no difference in total length of stay[35].

## Discussion

### Summary of evidence

Overall, this systematic review provides evidence that DDX generators have the potential to retrieve accurate diagnoses, albeit occasionally via lengthy lists. Consistent with previous reports the pooled accurate diagnosis retrieval rate of the DDX generators was 70%[11]. Commercially available DDX generators were associated with a higher accurate diagnosis retrieval rate (pooled rate = 81%) with some of the newer tools exhibiting the highest accurate diagnosis retrieval rates when compared to a gold standard. A small number of studies which compared the performance of DDX generators with the performance of primarily clinicians suggested that DDX generators were as likely as clinicians to include the correct diagnosis. Moreover, preliminary evidence from studies assessing accurate diagnosis retrieval by clinicians before and after the use of DDX generators indicated small but significant improvements in the ability of clinicians to assign the right diagnosis following the use of DDX generators.

The reporting of utility outcomes was variable. Breaking down utility data by DDX tool was not possible due to the low numbers of outcomes reported for some tools. Therefore the outcomes were combined across DDX tools for meaningful interpretation. This introduces error given the differences in DDX generators included which represent various iterations of tools over several years. Nevertheless, the majority of reported trends are consistent, albeit the heterogeneity prohibits calculating effect sizes with any certainty.

Although these findings are encouraging, they should be interpreted in the light of three key caveats, namely, that accurate diagnosis retrieval was assessed using a simplistic and unconventional manner (simple crude rates), the poor methodological quality of the included studies (including conflict of interest in some studies funded by DDX generator software manufacturers) and the high between-study heterogeneity. Key sources of the high levels of clinical heterogeneity were variations in the participants, cases and outcomes between studies. Studies included academics, students and clinicians of different grades and a mixture of real and simulated cases of varying complexities. Methodological heterogeneity was illustrated by the large variations in study designs and the high risk of bias demonstrated by some studies.

Unlike previous narrative reviews [11, 59] this is the first review which has been conducted using systematic methods including a meta-analysis which formally highlights a range of heterogeneity sources. We strongly recommend future high quality research in this area because no firm conclusions can be reached about the efficacy and utility of DDX generators based on the currently available evidence.

#### Diagnostic detail

Most DDX generators produce extensive lists. As a result the likelihood of having the ‘correct’ diagnosis listed increases but the value of the differential list to a clinician may decrease. Moreover, studies have demonstrated that it is possible for users to miss the correct diagnosis in a DDX output list and this effect is likely to be higher the longer the list is[23]. Such long lists may increase uncertainty in clinicians, which could prevent further uptake. Additionally, the relevance and comprehensiveness of DDX generator lists were generally low. This makes the task of identifying a correct or helpful diagnosis harder for busy clinicians.

#### Usage data

A reduction in the time taken to use the DDX generators was seen with newer tools such as Isabel and this may increase their acceptability in routine clinical practice. It is likely that advances in computer software designs and processing speeds in recent years have contributed to this. However, when DDX generator use was optional, the usage rates in two recent studies were generally low[22, 38]. Although there were a limited number of studies, it may suggest an element of unfamiliarity and scepticism from clinicians. Newer web-based interfaces of some tools such as Isabel may provide additional accessibility and improve future use as well their ability to utilise a wider set of remote databases leading to higher accurate diagnosis retrieval rates. Despite this, evidence of satisfaction levels in studies which ensured exposure to the tools, indicated a high level of satisfaction and this could be down to the novel experience as well as the potential benefits of using DDX generators[23, 30–32, 34, 43, 48].

#### Moderators of outcomes

The data suggested that junior members of the clinical team (e.g. medical students) inputted more data and were more likely to benefit from use of these tools. This is unsurprising given their lower levels of experience and this may have an important role to play in education and training in diagnostic techniques. Additionally, the relevance and accurate diagnosis retrieval rates of DDX generator outputs fell in the context of complex cases[22, 30, 33, 44], which is presumably when a DDX generator is most likely to be needed.

#### Outcomes

The evidence in relation to the number of investigations ordered and cost-effectiveness is limited by the small number of studies reporting relevant data. There was a trend towards use of DDX generators increasing the numbers of investigations requested[22, 29], but this was not seen consistently[32, 47]. Data on cost-effectiveness were also inconsistent[29, 35].

### Research and policy implications

This review demonstrates that DDX generators, particularly more contemporary versions have high accurate diagnosis retrieval rates when used in an experimental setting. The significance of this finding must be interpreted with caution however. As stated, the length of a DDX generator diagnostic list is a key predictor of accurate diagnosis retrieval. Long diagnostic lists are less specific and hence problematic for clinicians using these tools in a busy clinical setting. Moreover, the majority of DDX tools had no ability to rank the order of diagnoses and such a function is likely to have added value. Where rank/ordering of diagnoses were available, they were often limited to a small number of tools and varied significantly with their range.

Studies exploring the efficacy and utility of tools in prospective clinical settings are limited in number and quality. Recommendations are limited by the low quality of most included studies, their varying research designs, methodologies and heterogeneous outcomes.

Research should be conducted in a prospective, generalist clinical setting, ideally with cases stratified according to their complexity and users stratified according to their clinical experience. Given the heterogeneity and scarcity of high quality evidence we recommend a standardised and progressive approach (similar to that used by others such as the MRC complex interventions framework[60]) in developing DDX tools for use by clinicians in their routine clinical workflow. Although unlikely to be a priority for commercial entities, work must begin with understanding the barriers, facilitators and preferences to utilising DDX tools in routine clinical practice by professionals and they must have an understanding of patient perspectives. Such work should be followed by small scale exploratory studies such as controlled before-and-after studies exploring the impact on all relevant outcomes from diagnostic retrieval rates to the impact on ordering of tests/investigations and cost-effectiveness. We would recommend such studies receive funding independent of the software manufacturer to ensure scientific rigour.

Given the variance in the literature, where programmes are unable to rank diagnoses in order, at the least, developers should consider offering probabilities based on patient presentation, patient characteristics linked to electronic health records and potentially patient demographics. Where outcomes such as ranking are absent, we suggest standardisation of an “accurate retrieval” to be the correct diagnosis listed amongst the top-5 diagnoses produced. Following this, rigorous formal trials can be used to assess causal impact followed by pragmatic large-scale cost effectiveness studies with long enough follow-up periods to measure impact on patient safety, outcomes and costs.

At present, there is insufficient evidence to recommend the uptake of DDX tools in clinical settings. However, the data suggests a potential role for these tools in teaching diagnostic skills in a simulated setting.

### Strengths and Limitations

This systematic review had several strengths. The study eligibility criteria were broad to allow for a comprehensive overview of published data in this specialist area for generalist clinicians. The decision to include DDX tools that are no longer commercially available was helpful since much of the evidence relates to these, and much of the data relating to these is consistent with that found in more recent studies. Searches involved screening multiple (n = 16) databases supplemented by hand searches of the reference lists of studies included in the review. There is evidence that the non-inclusion of grey literature findings is associated with larger intervention effects[61]. To reduce this possibility in the case of DDX generators, grey literature was included in this review. Screening and data extraction were completed by two independent researchers and demonstrated very high levels of agreement. Exploratory work prior to the review allowed for a categorization of ‘utility’ concepts which matched the extracted data well. This review focused on both efficacy and utility because these are supplementary elements which determine the overall effectiveness of DDX tools.

Due to the nature of these tools and the low reported quality of the included studies there are limitations. Firstly, comparing the retrieval rates of different systems in different contexts using different cases is suboptimal resulting in our recommendation for future standardised work. The studies exhibited high heterogeneity and the main meta-analysis was based on pooling crude accurate diagnosis retrieval rates from studies lacking control groups. Although subgroup and sensitivity analyses were performed to explore key sources of heterogeneity (type of DDX generators, current availability and methodological quality), some subgroup analyses were based on a very small number of studies which do not allow the formation of robust conclusions. In addition, the high heterogeneity demonstrated by the meta-analysis could be attributed to other major variations such as study design and user and case characteristics which have not been accounted for in the analyses.

We pursued meta-analysis in this review because it facilitates the comparison of the results across studies, the examination of the consistency of effects, and the exploration of key variables that might account for inconsistencies[62]. A narrative synthesis does not allow such useful manipulations. In the light of the large between-study variations however, the findings of the meta-analysis should be interpreted with caution.

Some tools such as ILIAD[13, 15, 19–21, 36, 40, 42, 48, 51–53] and ISABEL[9, 22, 34, 37, 38, 45, 49, 55] was examined more often than others; some as low as once[9, 29, 44, 54, 56]. This made direct comparison between tools problematic. Furthermore the risk of bias assessment highlighted that some studies were partly funded by the DDX generator software manufacturers themselves, which is likely to introduce funder and publication bias. The significant risk of bias posed by this element requires careful consideration when interpreting the findings. Linked to this bias were concerns of the inconsistent and poor reporting of the data entry personnel, primarily whether they were funder employees, researchers or clinicians and whether they entered the data in real time or post-hoc.

Moreover, for the majority of studies, the accuracy of the systems was determined by measuring accurate diagnosis retrieval rates. In the absence of restrictions on the length of a diagnostic list, the clinical relevance of this concept is questionable. These retrieval rates were also based on the premise that gold standard clinician diagnoses are always correct and the review’s findings are heavily reliant on the internal validity of the primary studies. These issues are likely to impact on both the efficacy and utility of DDX generators. Whilst some of the older tools took longer to use, some of the newer tools demonstrated significantly lower time-taken to use. It is likely that improvements in information technology as well as advances between the interfaces of different health systems has contributed to the reduction in time and this may have important implications for use in routine clinical practice.

Another limitation of the study is that, although we went to extreme lengths to capture all relevant studies, it may be possible that DDX programmes exist on the market that have not been subject to peer-reviewed publications and as such have not been included in this review. Finally, a key barrier to the external validity of these results is that DDX generators were applied retrospectively across the majority of the studies. The tools must be used prospectively in order to reduce diagnostic error as part of a busy workload.

## Conclusions

Our findings demonstrate that DDX generators have the potential to improve diagnostic practice and thereby reduce diagnostic error, but there is currently insufficient evidence from the existing literature to recommend their routine use by clinicians. The literature is complex, with a variety of study designs, often of poor quality, used to appraise multiple iterations of different tools. While the pooled accurate diagnosis retrieval rates, particularly for the newer versions, are high; the review suggests that the benefit may be less in complex cases, when they are most likely to be used. Further prospective research is required to demonstrate clinical effectiveness.

### What is already known on this topic

Diagnostic errors commonly occur and can lead to preventable patient harm. Their causes are multifactorial and previous efforts to address diagnostic errors have focused on training clinicians to improve clinical and cognitive skills. Electronic DDX generators are evolving technologies which have the potential to reduce error by augmenting and influencing the diagnostic reasoning process of clinicians.

### What this study adds

Conclusions are limited by the heterogeneous study designs and poor study quality. DDX generators generally report high levels of accurate diagnosis retrieval but the clinical relevance of this term is questionable and there is no evidence that they perform significantly better than clinicians. However, DDX generators are not intended to replace clinicians and the relatively high levels of accurate diagnosis retrieval observed may augment the decision-making process of generalist clinicians. We also offer insights into what future studies should entail. Firstly, the integrity of the internal validity of studies must remain robust and primary studies must be conducted independently with no competing interests. Also, in addition to the efficacy of DDX tools, there are a number of important variables including the ranking of diagnoses, cost-effectiveness and time taken to use which should all be addressed in future research studies before further recommendations can be made.

## Supporting Information

S1 PRISMA Checklist PRISMA checklist. (DOC)

S1 File QUADAS-2 signalling questions. (DOCX)

S2 File Risk of bias of individual studies. (DOCX)

S3 File Utility data summary. (DOCX)
