# Supplementary material for: The Effectiveness of Electronic Differential Diagnoses (DDX) Generators: A Systematic Review and Meta-Analysis
Source: PLoS One. 2016 Mar 8;11(3):e0148991. doi: 10.1371/journal.pone.0148991 (PMC4782994; doi:10.1371/journal.pone.0148991)
Supplement: S1 File — (DOCX) [file pone.0148991.s002.docx]

S1 Appendix: Search strategy

1. DiagnosisPro.mp. [mp=ti, ab, tx, kw, ct, ot, sh, hw, bt, tn, dm, mf, dv, ac, de, md, sd, so, nm, kf, ps, rs, ui, tc, id, tm]
2. Diagnosis Pro [Including Limited Related Terms]
3. DxPlain [Including Limited Related Terms]
4. ISABEL.mp. [mp=ti, ab, tx, kw, ct, ot, sh, hw, bt, tn, dm, mf, dv, ac, de, md, sd, so, nm, kf, ps, rs, ui, tc, id, tm]
5. PEPID.mp. [mp=ti, ot, ab, tx, kw, ct, sh, de, md, sd, hw, bt, tn, dm, mf, dv, ac, ip, vo, pg, jn, yr, ey, cd, cl, pb, sa, ja, bd, dt, dp, pu, lp, pr, cp, rf, sj, ar, bs, cf, so, mo, op, os, pa, pi, pl, ry, st, nm, kf, ps, do, po, go, rs, an, ui, tc, id, tm]
6. VisualDx.mp. [mp=ti, ot, ab, tx, kw, ct, sh, de, md, sd, hw, bt, tn, dm, mf, dv, ac, ip, vo, pg, jn, yr, ey, cd, cl, pb, sa, ja, bd, dt, dp, pu, lp, pr, cp, rf, sj, ar, bs, cf, so, mo, op, os, pa, pi, pl, ry, st, nm, kf, ps, do, po, go, rs, an, ui, tc, id, tm]
7. couplers.mp. [mp=ti, ot, ab, tx, kw, ct, sh, de, md, sd, hw, bt, tn, dm, mf, dv, ac, ip, vo, pg, jn, yr, ey, cd, cl, pb, sa, ja, bd, dt, dp, pu, lp, pr, cp, rf, sj, ar, bs, cf, so, mo, op, os, pa, pi, pl, ry, st, nm, kf, ps, do, po, go, rs, an, ui, tc, id, tm]
8. DDx [Including Limited Related Terms]
9. D/Dx [Including Limited Related Terms]
10. Differential diagnosis generator* [Including Limited Related Terms]
11. Clinical decision support system [Including Limited Related Terms]
12. Computer assisted diagnosis [Including Limited Related Terms]
13. web-based.mp. [mp=ti, ot, ab, tx, kw, ct, sh, de, md, sd, hw, bt, tn, dm, mf, dv, ac, ip, vo, pg, jn, yr, ey, cd, cl, pb, sa, ja, bd, dt, dp, pu, lp, pr, cp, rf, sj, ar, bs, cf, so, mo, op, os, pa, pi, pl, ry, st, nm, kf, ps, do, po, go, rs, an, ui, tc, id, tm]
14. internet-based.mp. [mp=ti, ot, ab, tx, kw, ct, sh, de, md, sd, hw, bt, tn, dm, mf, dv, ac, ip, vo, pg, jn, yr, ey, cd, cl, pb, sa, ja, bd, dt, dp, pu, lp, pr, cp, rf, sj, ar, bs, cf, so, mo, op, os, pa, pi, pl, ry, st, nm, kf, ps, do, po, go, rs, an, ui, tc, id, tm]
15. computer-based.mp. [mp=ti, ot, ab, tx, kw, ct, sh, de, md, sd, hw, bt, tn, dm, mf, dv, ac, ip, vo, pg, jn, yr, ey, cd, cl, pb, sa, ja, bd, dt, dp, pu, lp, pr, cp, rf, sj, ar, bs, cf, so, mo, op, os, pa, pi, pl, ry, st, nm, kf, ps, do, po, go, rs, an, ui, tc, id, tm]
16. programme-based.mp. [mp=ti, ot, ab, tx, kw, ct, sh, de, md, sd, hw, bt, tn, dm, mf, dv, ac, ip, vo, pg, jn, yr, ey, cd, cl, pb, sa, ja, bd, dt, dp, pu, lp, pr, cp, rf, sj, ar, bs, cf, so, mo, op, os, pa, pi, pl, ry, st, nm, kf, ps, do, po, go, rs, an, ui, tc, id, tm]
17. application-based.mp. [mp=ti, ot, ab, tx, kw, ct, sh, de, md, sd, hw, bt, tn, dm, mf, dv, ac, ip, vo, pg, jn, yr, ey, cd, cl, pb, sa, ja, bd, dt, dp, pu, lp, pr, cp, rf, sj, ar, bs, cf, so, mo, op, os, pa, pi, pl, ry, st, nm, kf, ps, do, po, go, rs, an, ui, tc, id, tm]
18. 1 or 2 or 3 or 4 or 5 or 6 or 7 or 8 or 9 or 10 or 11 or 12 or 13 or 14 or 15 or 16 or 17
19. Diagnosis [Including Limited Related Terms]
20. Diagnoses [No Related Terms]
21. Diagnostic [No Related Terms]
22. Diagnosing [No Related Terms]
23. Delayed diagnosis [Including Limited Related Terms]
24. wrong diagnosis [No Related Terms]
25. Late diagnosis.mp. [mp=ti, ot, ab, tx, kw, ct, sh, de, md, sd, hw, bt, tn, dm, mf, dv, ac, ip, vo, pg, jn, yr, ey, cd, cl, pb, sa, ja, bd, dt, dp, pu, lp, pr, cp, rf, sj, ar, bs, cf, so, mo, op, os, pa, pi, pl, ry, st, nm, kf, ps, do, po, go, rs, an, ui, tc, id, tm]
26. Missed diagnosis.mp. [mp=ti, ot, ab, tx, kw, ct, sh, de, md, sd, hw, bt, tn, dm, mf, dv, ac, ip, vo, pg, jn, yr, ey, cd, cl, pb, sa, ja, bd, dt, dp, pu, lp, pr, cp, rf, sj, ar, bs, cf, so, mo, op, os, pa, pi, pl, ry, st, nm, kf, ps, do, po, go, rs, an, ui, tc, id, tm]
27. Diagnostic error.mp. [mp=ti, ot, ab, tx, kw, ct, sh, de, md, sd, hw, bt, tn, dm, mf, dv, ac, ip, vo, pg, jn, yr, ey, cd, cl, pb, sa, ja, bd, dt, dp, pu, lp, pr, cp, rf, sj, ar, bs, cf, so, mo, op, os, pa, pi, pl, ry, st, nm, kf, ps, do, po, go, rs, an, ui, tc, id, tm]
28. Early diagnosis [No Related Terms]
29. Diagnostic accuracy [No Related Terms]
30. Differential diagnosis [Including Limited Related Terms]
31. 19 or 20 or 21 or 22 or 23 or 24 or 25 or 26 or 27 or 28 or 29 or 30
32. 18 and 31
33. remove duplicates from 32
