# Supplementary material for: The Effectiveness of Electronic Differential Diagnoses (DDX) Generators: A Systematic Review and Meta-Analysis
Source: PLoS One. 2016 Mar 8;11(3):e0148991. doi: 10.1371/journal.pone.0148991 (PMC4782994; doi:10.1371/journal.pone.0148991)
Supplement: S2 File — (DOCX) [file pone.0148991.s003.docx]

| **STUDY ID** | **RISK OF BIAS** | | | | | **APPLICABILITY** | | | **TOTAL** |
| --- | --- | --- | --- | --- | --- | --- | --- | --- | --- |
|  | **Risk of bias** | | | | | **Applicability concerns** | | |  |
|  | **Case selection** | **Index test** | **Reference standard** | **Analysis of all cases** | **Commercial funding** | **Case selection** | **Index test** | **Reference standard** |  |
| **Apkon 2005** | + | - | + | - | + | + | - | + | **5** |
| **Arene 1998** | - | - | - | + | ? | + | - | + | **3** |
| **Bacchus 1994** | + | - | + | + | + | + | - | - | **5** |
| **Bankowitz 1989** | - | - | - | - | + | + | + | + | **4** |
| **Bavdekar 2005** | + | + | + | - | + | - | - | + | **5** |
| **Berner 1994** | - | - | + | - | + | + | - | + | **4** |
| **Berner 1999** | - | - | + | - | + | + | - | + | **4** |
| **Bond 2011** | + | + | + | + | + | + | - | + | **7** |
| **Carlson 2011** | - | + | - | - | + | + | - | + | **4** |
| **Elkin 2010** | + | + | + | + | + | + | - | + | **7** |
| **Elstein 1996** | - | - | + | + | + | + | - | + | **5** |
| **Feldman 1991** | - | ? | - | - | - | + | - | + | **2** |
| **Friedman 1999** | - | - | + | - | - | + | - | + | **2** |
| **Gozum 1994** | + | - | + | + | ? | - | - | + | **4** |
| **Graber 2003** | - | - | - | + | + | + | - | + | **4** |
| **Graber 2008** | + | + | + | + | + | + | - | + | **7** |
| **Graber 2009** | - | + | - | + | + | + | - | + | **5** |
| **Hammersley 1988** | + | - | + | + | ? | + | - | + | **5** |
| **Heckerling 1991** | - | - | + | + | + | + | - | + | **5** |
| **Lange 1997** | - | - | + | + | ? | + | - | + | **4** |
| **Lau 1995** | - | - | - | - | + | - | - | + | **2** |
| **Lemaire 1999** | - | - | + | + | + | + | - | + | **5** |
| **Li 1995** | - | - | + | - | + | + | - | + | **4** |
| **Lincoln 1991** | - | - | - | + | ? | + | - | + | **3** |
| **Miller 1982** | - | - | + | - | ? | + | - | + | **3** |
| **Miller 1986** | + | - | - | - | ? | + | - | + | **3** |
| **Murphy 1996** | - | - | - | + | ? | + | + | + | **4** |
| **Nelson 1985** | + | - | - | + | ? | + | - | + | **4** |
| **Ramnarayan 2003 (Pt. 1)** | - | ? | - | + | ? | + | - | + | **3** |
| **Ramnarayan 2003 (Pt. 2)** | - | ? | + | - | ? | + | - | + | **3** |
| **Ramnarayan and Roberts 2006** | - | ? | - | - | - | + | - | + | **2** |
| **Ramnarayan and Winrow 2006** | - | + | + | - | - | + | + | + | **5** |
| **Ramnarayan 2007** | - | + | - | - | + | + | - | + | **4** |
| **Rodriguez-Gonzalez 2012** | - | ? | - | + | ? | + | - | + | **3** |
| **Waxman 1990 (retrospective)** | - | - | + | + | ? | + | - | + | **4** |
| **Waxman 1990 (prospective)** | - | - | + | + | ? | + | - | + | **4** |
| **Wexler 1975** | - | - | + | + | ? | + | + | + | **5** |
| **Wolf 1997** | - | - | + | + | + | + | - | + | **5** |

**S2 Appendix: Risk of bias of individual studies**
