# Supplementary material for: The Effectiveness of Electronic Differential Diagnoses (DDX) Generators: A Systematic Review and Meta-Analysis
Source: PLoS One. 2016 Mar 8;11(3):e0148991. doi: 10.1371/journal.pone.0148991 (PMC4782994; doi:10.1371/journal.pone.0148991)
Supplement: S3 File — (DOCX) [file pone.0148991.s004.docx]

S3 Appendix. Utility data summary

| **DDX TOOL** | **OUTCOME** | **DETAILS** | **STUDY** |
| --- | --- | --- | --- |
| **DxPLAIN** | **Comprehensiveness** | Proportion of correct diagnoses present in DDX knowledge base (KB): | **Berner 1994** |
|  |  | •       **DxPLAIN** = 91% (95%CI; 86-97%) |  |
|  |  |  |  |
|  |  | Assigned ‘comprehensiveness’ scores based on proportion of appropriate diagnoses agreed by a group of experts which appeared on the DDX differential list (for cases present in KB): |  |
|  |  | •       **Dxplain** = 38% (95% CI; 33%-44% |  |
|  | **Cost-effectiveness** | Average total admission costs significantly reduced in DxPlain group compared to control group: | **Elkin 2000** |
|  |  | •       Total costs: DxPlain = $7328; Control = $8318; 11.9% reduction (95% CI; 3.7-19.5%); p=0.0012 |  |
|  |  | No significant difference in length of stay in DxPlain group compared to control group: |  |
|  |  | •       Length of stay: Dx Plain = 3.99 days; Control = 4.14; 3.62% reduction; p=0.89; 11.9% (3.7-9.5%) reduction in total costs in intervention group (p=0.0012). Difference in means = $990 |  |
|  | **Diagnostic list** | The average number of appropriate diagnoses as judged by the expert panel increased following consultation with all DDX tools: | **Berner 1994** |
|  |  | •       **DxPLAIN** 2.6 extra diagnoses per case (95% CI; 2.0-3.1) |  |
|  | **Number of diagnoses** | Mean 46.5 diagnostic suggestions per case: | **Hammersley 1988** |
|  |  | •       **DxPLAIN** = 57.3 |  |
|  | **Relevance** | Average ranking of correct diagnosis | **Hammersley 1988** |
|  |  | •       **DxPLAIN** = 10.7 (range 1-54) |  |
|  |  | Assigned ‘relevance’ scores based on proportion of DDX tool-generated diagnoses felt to be appropriate by a group of experts: | **Berner 1994** |
|  |  | •       **DxPLAIN** = 26% (95% CI; 23-29%) |  |
|  | **Time to use** | Average time to use, per case: | **Hammersley 1988** |
|  |  | •       **DxPLAIN**: data abstraction – 20 minutes; data entry = 9.3 minutes |  |
| **ILIAD** | **Diagnostic list** | Changes to diagnostic list following ILIAD consultation: | **Murphy 1996** |
|  |  | •       Diagnoses added: Mean 1.3 diagnoses per case |  |
|  |  | •       Diagnoses reordered: Mean 0.3 diagnoses per case |  |
|  |  | Average changes to diagnostic list following DDX tool consultation: | **Friedman 1999** |
|  |  | •       **ILIAD**: Correct diagnosis added = 10.4%; Correct diagnosis removed = 6.3% |  |
|  | **Relevance** | Average ranking of correct diagnosis: | **Heckerling 1991** |
|  |  | •       Overall = 2.3 |  |
|  |  | •       Pre-consultation = 2.0 (n=28) |  |
|  |  | •       Post-consultation = 1.5 (n=22), p<0.008 |  |
|  |  | Assigned ‘relevance’ scores based on proportion of DDX tool-generated diagnoses felt to be appropriate by a group of experts: | **Berner 1994** |
|  |  | •       **ILIAD** = 21% (95% CI; 17-24%) |  |
|  |  | Mean proportion of diagnoses appearing within top 6 of ILIAD output = 20% | **Murphy 1996** |
|  |  | Change in DQS following DDX tool consultation: | **Friedman 1999** |
|  |  | •       **ILIAD** = before 5.7 (5.4-6.0); after 6.0 (5.7-6.3); effect size 0.20 (0.08-0.32); p<0.001 |  |
|  |  | Proportion of diagnoses appearing within top 5 or top 10 or the DDX output: | **Graber 2003** |
|  |  | •       **ILIAD**: Top 5 = 36%; top 10 = 51% |  |
|  | **Satisfaction** | Users more likely to change diagnosis if felt ILIAD output was helpful (25.3%) rather than not helpful (21.3%) | **Wolf 1997** |
|  |  | Users less likely to change diagnosis if they had already acknowledged need to seek diagnostic consultation prior to using ILIAD (19.4%) compared to those who did not plan to seek alternative diagnostic consultation (22.6%) |  |
| **ILIAD** | **Clinical experience** | **Changes to diagnostic list following ILIAD consultation**: | **Murphy 1996** |
|  |  | •       Diagnoses added: Medical students = 2.4 diagnoses per case; Residents = 0.85; Attending physicians = 0.54 (ANOVA: p=0.0001) |  |
|  |  | •       Diagnoses reordered: Medical students = 0.30 diagnoses per case; Residents = 0.42; Attending Physicians = 0.27 (ANOVA: p=0.39) |  |
|  |  | •       Pre-consultation correct diagnoses: Medical students = 22%; Residents = 50%; Attending Physicians = 54% (ANOVA: p=0.0003) |  |
|  |  | •       Post-consultation correct diagnoses: Medical students = 31%; Residents = 55%; Attending Physicians = 59% (ANOVA: p=0.004) |  |
|  |  |  |  |
|  |  | **Diagnostic accuracy:** |  |
|  |  | •       Proportion of correct diagnoses appearing anywhere in ILIAD output: Medical students = 40.8%; Residents = 46.3%; Attending Physicians= 49.3% (P=0.37) |  |
|  |  | •       Proportion of correct diagnoses appearing within top 6 of ILIAD output: Medical students = 19%; Residents = 17.6%; Attending Physicians= 25% (P=0.41) |  |
|  |  | Impact of previous case exposure improved diagnostic accuracy using Iliad among nursing students for chest pain cases (r=0.40, p<0.10), but not abdominal pain cases (r=-0.15, p>0.10) | **Lange 1997** |
|  | **Comprehensiveness** | 56% of correct diagnoses present in Iliad’s KB | **Heckerling 1991** |
|  |  | Proportion of correct diagnoses present in DDX knowledge base (KB): | **Berner 1994** |
|  |  | •       **ILIAD** = 76% (95% CI; 68%-85%) |  |
|  |  |  |  |
|  |  | Assigned ‘comprehensiveness’ scores based on proportion of appropriate diagnoses agreed by a group of experts which appeared on the DDX differential list (for cases present in KB): |  |
|  |  | •       **ILIAD** = 27% (95% CI; 22%-32%) |  |
|  | **Diagnostic list** | The average number of appropriate diagnoses as judged by the expert panel increased following consultation with all DDX tools: | **Berner 1994** |
|  |  | •       **ILIAD** = 2.2 (95% CI; 1.7-2.8) |  |
|  |  | In ‘over 70%’ of cases, consultation with ILIAD had no effect on the accuracy of clinicians; in 35% of these cases ILIAD displayed the correct diagnosis but clinicians ignored it | **Elstein 1996** |
|  |  | Quality of diagnostic list judged to have improved in 15% of cases with Attending physicians and Medical students but no improvement was seen for residents. Quality declined in 12% of cases overall (Attending physicians = 9%; Residents = 17%; Medical students = 13%) |  |
| **ISABEL** | **Case difficulty** | Greater reduction in diagnostic errors of omission noted for ‘difficult’ cases rather than ‘easy’ cases (non-significant) | **Ramnarayan and Roberts 2006** |
|  | **Clinical experience** | Medical students reported greatest increase in diagnostic list length (2.6 diagnoses) after ISABEL consultation, compared to 1.4 extra diagnoses for Consultants. | **Ramnarayan and Roberts 2006** |
|  |  | Medical students demonstrated greatest reduction in diagnostic errors of omission post-ISABEL use (-0.69); Senior house officers (SHOs) had the smallest reduction (0.3). |  |
|  |  | No significant change in diagnostic quality score post-ISABEL consultation between grades (medical student, SHO, Registrar, Consultant) |  |
|  |  | Medical students were added significantly more irrelevant diagnoses (1.1 per case) after ISABEL consultation compared to Consultants (0.3 per case); p<0.01) |  |
|  |  | Greater improvement in Diagnostic Quality Score seen for SHOs (8.3 [SD 11.6]) than Registrars (3.8[SD 6.1]) | **Ramnarayan and Winrow 2006** |
|  |  | Students with lowest pre-ISABEL diagnostic accuracy score (DAS) benefitted most (-0.67 correlation between pre-DAS and magnitude of diagnostic improvement) | **Carlson 2011** |
|  | **Comprehensiveness** | 87% of correct stage 2 diagnoses present in ISABEL’s KB (but ‘synonymous’ diagnoses presented in 77% of these | **Ramnarayan 2003** |
|  | **Diagnostic list** | Changes to diagnostic list following ISABEL consultation: | **Ramnarayan and Roberts 2006** |
|  |  | •       Length of list: increased from 3.9 to 5.7 diagnoses post-ISABEL |  |
|  |  | •       Addition of 'clinically important diagnosis' in 12.5% cases (95% CI 10.1-14.9) |  |
|  |  | •       No clinically significant diagnoses were deleted |  |
|  |  | Average number of diagnoses following ISABEL consultation increased from 2.2 to 3.2 (no significance data) | **Ramnarayan and Winrow 2006** |
|  |  | In 5/104 cases, the correct diagnosis was prompted by ISABEL but ignored by clinicians |  |
|  |  | Average reduction in unsafe diagnoses: Pre-SABEL 0.49; Post-ISABEL 0.32 (p<0.001) |  |
|  | **Frequency of use** | 54% (n=52) of clinician completed all 12 allocated cases 52/97 clinicians completed 12 cases | **Ramnarayan and Roberts 2006** |
|  |  | 7.9% (n=6) of clinicians used ISABEL regularly (>once/week) |  |
|  |  | 56% (n=33) of medical students randomised to have access to ISABEL used it | **Graber 2009** |
|  | **Investigations** | At least one significant investigation was added to management plan in 9.3% (n=70) of cases | **Ramnarayan and Roberts 2006** |
|  |  | Average number of tests increased from 2.7 to 2.9 following ISABEL consultation (no significance data) | **Ramnarayan and Winrow 2006** |
| **ISABEL** | **Number of diagnoses** | Mean 13 diagnostic suggestions per case | **Ramnarayan 2003** |
|  |  | Mean 30 diagnostic suggestions per case | **Graber 2009** |
|  | **Relevance** | Following use of ISABEL the Mean Quality Score applied to the diagnostic list increased from 0.383 to 0.426; mean change 0.044 (95% CI; 0.032-0.054, P<0.001) | **Ramnarayan and Roberts 2006** |
|  |  |  |  |
|  |  | Number of irrelevant diagnoses increased by 0.7 (95% CI; 0.5-0.75) from 0.7 per case to 1.4 per case following use of ISABEL |  |
|  |  | Correct diagnosis appeared in top ten of ISABEL output in 78% of cases | **Ramnarayan 2007** |
|  |  | Overall diagnostic quality score (DQS) increased by 6.9 (S.D.) following ISABEL consultation | **Ramnarayan 2007** |
|  |  | Medical students who chose to use ISABEL were more likely to o have a correct first choice diagnosis 61% than non-users (40%); p=0.064 | **Graber 2009** |
|  | **Satisfaction** | Junior doctor mean satisfaction scores for ISABEL: | **Ramnarayan and Winrow 2006** |
|  |  | •       Patient management = 32% (95% CI; 28%-39%); n=125 |  |
|  |  | •       Educational adjunct = 48% (95% CI; 40%-56%) |  |
|  |  | Medical student experience of ISABEL: | **Carlson 2011** |
|  |  | •       68% reported information generated helped to confirm their own diagnostic list |  |
|  |  | •       77% suggested that Isabel suggested diagnostic options that might have been missed |  |
|  |  | •       77% reported that diagnostic options provided did not include diagnoses they expected to see |  |
|  |  | •       91% agreed that it was easy to use |  |
|  |  | •       73% would have preferred access earlier on in their education. |  |
|  | **Time to use** | Average time to use, per case (n=633): | **Ramnarayan and Roberts 2006** |
|  |  | •       Pre-ISABEL use = 6 minutes 2 seconds |  |
|  |  | •       Post-ISABEL use = 1 minute |  |
|  |  | Median usage time 98 seconds (IQR = 50-201 seconds) | **Ramnarayan and Winrow 2006** |
|  |  | Less than one minute (manual entry) per case | **Graber 2008** |
| **MEDITEL** | **Comprehensiveness** | Proportion of correct diagnoses present in DDX knowledge base (KB): | **Berner 1994** |
|  |  | •       **MEDITEL** = 85% (95% CI; 78-92%) |  |
|  |  |  |  |
|  |  | Assigned ‘comprehensiveness’ scores based on proportion of appropriate diagnoses agreed by a group of experts which appeared on the DDX differential list (for cases present in KB): |  |
|  |  | •       **Meditel** = 39% (95% CI; 32%-46%) |  |
|  | **Diagnostic list** | The average number of appropriate diagnoses as judged by the expert panel increased following consultation with all DDX tools: | **Berner 1994** |
|  |  | •       **MEDITEL** = 2.2 (95% CI; 1.8-2.5) |  |
|  | **Number of diagnoses** | Mean 40.6 diagnostic suggestions per case | **Wexler 1975** |
|  |  | Mean 46.5 diagnostic suggestions per case: | **Hammersley 1988** |
|  |  | •       **MEDITEL** = 46.5 |  |
|  | **Relevance** | Average ranking of correct diagnosis | **Hammersley1988** |
|  |  | •       **MEDITEL** = 9 (range 1-50) |  |
|  |  | **RETROSPECTIVE STUDY** | **Waxman 1990** |
|  |  | •       Average ranking of correct diagnosis was 10^th^ |  |
|  |  | • |  |
|  |  | **PROSPECTIVE STUDY** |  |
|  |  | •       47.1% (n=51) of correct diagnoses listed in top 5 |  |
|  |  | •       56.9% (n=51) of correct diagnoses listed in top 10 |  |
|  |  | Assigned ‘relevance’ scores based on proportion of DDX tool-generated diagnoses felt to be appropriate by a group of experts: | **Berner 1994** |
|  |  | •       **MEDITEL** = 23% (95% CI; 20-26%) |  |
|  | **Time to use** | Average time to use, per case: | **Hammersley 1988** |
|  |  | •       **MEDITEL**: data abstraction = 20 minutes; data entry = 1-2 minutes |  |
| **Problem-knowledge couplers** | **Cost-effectiveness** | Average cost of laboratory testing, diagnostic imaging and total resource consumption higher in group randomised to PKC: | **Apkon 2005** |
|  |  | •       Total resource consumption: PKC = $789 (IQR 375-1654); Control = $698 (340-1530); p=0.05 |  |
|  |  | •       Laboratory testing: PKC = $43 [IQR 0-144]; Control = $31 [0-139]; p=0.04 |  |
|  |  | •       Diagnostic imaging: PKC = $31 (IQR 0-148); Control = $29 (0-127); p=0.26 |  |
| **QMR** | **Case difficulty** | QMR accuracy reduced in cases involving multiple organ systems. For ‘highly complex cases’, accuracy with QMR was 45% compared to 79.5% clinician accuracy | **Arene 1998** |
|  |  | Authors note QMR had difficulty combining results from different systems |  |
|  |  | Mean accuracy using QMR greater for ‘easy’ cases (67%) compared to ‘difficult’ cases 32% (p<0.01) | **Berner 1999** |
|  | **Comprehensiveness** | 83% of correct diagnoses present in QMR’s KB | **Miller 1986** |
|  |  | Proportion of correct diagnoses present in DDX knowledge base (KB): | **Berner 1994** |
|  |  | •       **QMR** = 73% (95% CI; 65-82%) |  |
|  |  | Assigned ‘comprehensiveness’ scores based on proportion of appropriate diagnoses agreed by a group of experts which appeared on the DDX differential list (for cases present in KB): |  |
|  |  | •       **QMR** = 30% (95% CI; 25-35%) |  |
|  |  | Mean ‘comprehensiveness’ (defined as per Berner 1994) for QMR was 22% (S.D. 0.06) | **Berner 1999** |
|  |  | 89% of correct diagnoses present in QMR’s KB | **Lemaire 1999** |
|  | **Diagnostic list** | Changes to diagnostic list following QMR-consultation (in cases where QMR/clinician agreement): | **Miller 1986** |
|  |  | •       Diagnoses added = 9.5% (n=21) |  |
|  |  | •       Diagnoses removed = 9.5% (n=21) |  |
|  |  | •       Diagnoses reordered = 9.5% (n=21) |  |
|  |  | Clinicians more likely to add rather than exclude diagnoses following QMR consultation: | **Bankowitz 1989** |
|  |  | •       QMR diagnosis added: Ward team = 45% of consultations; Consultants = 48% |  |
|  |  | •       Non-QMR diagnosis added: Ward team = 23%; Consultants = 0% |  |
|  |  | •       Diagnosis excluded: Ward team = 26%; Consultants = 19% |  |
|  |  | •       Diagnosis reordered: Ward team = 23%; Consultants = 10% |  |
|  |  | The average number of appropriate diagnoses as judged by the expert panel increased following consultation with all DDX tools: | **Berner 1994** |
|  |  | •       **QMR** = 2.0 (95% CI; 1.4-2.5) |  |
|  |  | Average changes to diagnostic list following DDX tool consultation: | **Friedman 1999** |
|  |  | •       **QMR**: Correct diagnosis added = 13.6%; Correct diagnosis removed = 5.8% |  |
| **QMR** | **Number of diagnoses** | ‘As many as 15 diagnoses’ suggested by QMR per case | **Bankowitz 1989** |
|  |  | Mean 48.5 diagnostic suggestions per case | **Arene 1998** |
|  |  | Some searches generated over 150 results | **Lemaire 1999** |
|  | **Relevance** | Correct diagnosis highest ranked diagnosis per group: | **Bankowitz 1989** |
|  |  | •       QMR 60% (n=12) |  |
|  |  | •       Ward team 30% (n=6) |  |
|  |  | •       Consultants 50% (n=10) |  |
|  |  | Assigned ‘relevance’ scores based on proportion of DDX tool-generated diagnoses felt to be appropriate by a group of experts: | **Berner 1994** |
|  |  | •       **QMR** = 46% (95% CI; 39-54%) |  |
|  |  | Mean ‘comprehensiveness’ (as defined by Berner 1994) for QMR was 61% (S.D. 0.14) | **Berner 1999** |
|  |  | Comprehensiveness scores were significantly higher for ‘easy’ (67%; S.D. 0.18) compared to ‘difficult’ cases (56%; S.D. 0.15); p<0.01 and also for ‘high information quality’ (68%; S.D. 0.15) compared to ‘low information quality’ cases (55%; S.D. 0.18); p<0.01 |  |
|  |  | Change in DQS following DDX tool consultation: | **Friedman 1999** |
|  |  | •       **QMR** = before 5.6 (5.3-5.9); after 6.2 (5.9-6.5); effect size 0.45 (0.31-0.59); p<0.001 |  |
|  |  | Correct diagnosis ranked highest in 23% of cases | **Lemaire 1999** |
|  |  | Proportion of diagnoses appearing within top 5 or top 10 or the DDX output: | **Graber 2003** |
|  |  | •       **QMR**: Top 5 = 32% of correct diagnoses; top 10=44% of correct diagnoses |  |
|  |  | •       **ILIAD**: Top 5 = 36%; top 10 = 51% |  |
| **QMR** | **Satisfaction** | Ward team experience of QMR: | **Miller 1986** |
|  |  | •       'Very helpful' in 33.3% (n=36) |  |
|  |  | •       'Helpful' in 39% |  |
|  |  | •       'Unhelpful/misleading' in 6% |  |
|  |  | •       ‘Incorrect’ in 22% |  |
|  |  | Ward team experience of QMR: | **Bankowitz 1989** |
|  |  | •       Educational value |  |
|  |  | o   Helpful n=25 (81%) |  |
|  |  | o   Neutral n=3 (10%) |  |
|  |  | o   Not helpful (n=1) 3% |  |
|  |  | •       Patient management |  |
|  |  | o   Helpful (n=25) 81% |  |
|  |  | o   Neutral n=5 (16%) |  |
|  |  | o   Not helpful 0% |  |
|  |  | Intern experience of QMR: | **Bacchus 1994** |
|  |  | •       ‘helpful in arriving at a provisional diagnosis' n=14 (88%) |  |
|  |  | •       ‘helpful at formulating a differential diagnosis' n=15 (94%) |  |
|  |  | •       'helpful in choosing useful investigations' n=12 (75%) |  |
|  |  | Chief resident experience of QMR: | **Arene 1998** |
|  |  | •       helped increase 'understanding in disease processes' in 55% |  |
|  |  | •       helped focus on pertinent clinical data in 53% |  |
|  |  | •       rated not helpful in 15% of cases |  |
|  | **Time to use** | Time for ‘full diagnostic analysis’ ranged between 1-4 hours per case | **Bankowitz 1989** |
| **RECONSIDER** | **Case difficulty** | Average accuracy based on number of diagnoses per case: | **Nelson 1985** |
|  |  | •       1 diagnosis = 67% (n=218) |  |
|  |  | •       2 diagnoses = 62% (n=216) |  |
|  |  | •       3 diagnoses = 42% (n=55) |  |
|  | **Relevance** | Correct diagnosis ranked first in RECONSIDER output in 28% of cases (n=105) and in the top 10 in 68% of cases (n=105) | **Nelson 1985** |
